# Dysfunction of NMDA receptors in neuronal models of an autism spectrum disorder patient with a *DSCAM* mutation and in *Dscam-*knockout mice

**DOI:** 10.1038/s41380-021-01216-9

**Published:** 2021-07-12

**Authors:** Chae-Seok Lim, Min Jung Kim, Ja Eun Choi, Md Ariful Islam, You-Kyung Lee, Yinyi Xiong, Kyu-Won Shim, Jung-eun Yang, Ro Un Lee, Jiah Lee, Pojeong Park, Ji-Hye Kwak, Hyunhyo Seo, Chul Hoon Kim, Jae-Hyung Lee, Yong-Seok Lee, Su-Kyeong Hwang, Kyungmin Lee, Jin-A Lee, Bong-Kiun Kaang

**Affiliations:** 1 School of Biological Sciences, Seoul National University, Seoul, 08826 South Korea; 2 Department of Pharmacology, Wonkwang University School of Medicine, Jeonbuk, 54538 South Korea; 3 Department of Biotechnology and Biological Sciences, Hannam University, Daejeon, 34430 South Korea; 4 Interdisciplinary Program in Bioinformatics, Seoul National University, Seoul, 08826 South Korea; 5 Department of Anatomy, School of Medicine, Kyungpook National University, Daegu, 41944 South Korea; 6 Department of Pharmacology, Yonsei University College of Medicine, Seoul, 03722 South Korea; 7 Department of Life and Nanopharmaceutical Sciences, Department of Oral Microbiology, Kyung Hee University School of Dentistry, Seoul, 02447 South Korea; 8 Department of Physiology, Biomedical Sciences, Neuroscience Research Institute, Seoul National University College of Medicine, Seoul, 03080 South Korea; 9 Department of Pediatrics, School of Medicine, Kyungpook National University, Daegu, 41944 South Korea; 10 Present Address: Department of Pharmaceutical Sciences, School of Health and Life Sciences, North South University, Dhaka-1229, Bangladesh

**Keywords:** Stem cells, Autism spectrum disorders

## Abstract

Heterogeneity in the etiopathology of autism spectrum disorders (ASD) limits the development of generic remedies, requires individualistic and patient-specific research. Recent progress in human-induced pluripotent stem cell (iPSC) technology provides a novel platform for modeling ASDs for studying complex neuronal phenotypes. In this study, we generated telencephalic induced neuronal (iN) cells from iPSCs derived from an ASD patient with a heterozygous point mutation in the *DSCAM* gene. The mRNA of DSCAM and the density of DSCAM in dendrites were significantly decreased in ASD compared to control iN cells. RNA sequencing analysis revealed that several synaptic function-related genes including NMDA receptor subunits were downregulated in ASD iN cells. Moreover, NMDA receptor (R)-mediated currents were significantly reduced in ASD compared to control iN cells. Normal NMDA-R-mediated current levels were rescued by expressing wild-type DSCAM in ASD iN cells, and reduced currents were observed by truncated DSCAM expression in control iN cells. shRNA-mediated DSCAM knockdown in control iN cells resulted in the downregulation of an NMDA-R subunit, which was rescued by the overexpression of shRNA-resistant DSCAM. Furthermore, DSCAM was co-localized with NMDA-R components in the dendritic spines of iN cells whereas their co-localizations were significantly reduced in ASD iN cells. Levels of phospho-ERK1/2 were significantly lower in ASD iN cells, suggesting a potential mechanism. A neural stem cell-specific *Dscam* heterozygous knockout mouse model, showing deficits in social interaction and social memory with reduced NMDA-R currents. These data suggest that *DSCAM* mutation causes pathological symptoms of ASD by dysregulating NMDA-R function.

## Introduction

Autism spectrum disorder (ASD) refers to a group of complex neurodevelopmental disorders characterized by persistent difficulties with social communication and social interaction, restricted and repetitive behavior (including sensory behaviors), activities, or interests, and early childhood manifestation of symptoms [1] and poses a serious threat to a patient’s ability to harmoniously adapt to society. Although ASD is clearly defined by diagnostic criteria, the levels of severity and penetration may vary among individuals. The complexity and heterogeneity of ASD pathogenesis, along with the lack of a proper human cellular model system, makes it difficult to explore the pathophysiological mechanisms of ASD in human development. Nonetheless, recent progress in human-induced pluripotent stem cell (iPSC) technology provides a novel platform for modeling neurodevelopmental or psychiatric disorders in patients with monogenic or idiopathic mutations, and provides access to patient-specific cells for drug discovery and personalized medicine [2, 3]. Moreover, recent reports highlight the rational use of iPSC-derived neurons to model ASDs in the study of complex neuronal phenotypes [4, 5]. Genes for many synaptic proteins, such as *Shanks*, have been identified as ASD candidate genes, paving the way for studies on ASD pathogenesis using cellular and animal models [6–9]. Among the synaptic proteins associated with ASD, N-methyl-D-aspartate receptor (NMDA) receptors (Rs) have garnered attention because many ASD models with mutations in distinct genes show NMDA-R dysfunctions [10–13] and NMDA-Rs are important in neuronal differentiation and synaptic plasticity in several brain regions including the hippocampus and cortex [14, 15].

Variants in the *Down syndrome cell adhesion molecule* (*DSCAM*) gene exist in many ASD patients [16–20], suggesting that *DSCAM* is an ASD-related gene. DSCAM has been implicated in the development of the human central and peripheral nervous system in the context of axon targeting, dendrite branching, and synapse formation [21, 22]. DSCAM is involved in self-avoidance, a mechanism in which the neuronal processes repel each other during dendritic arborization and axonal branching for uniform distribution of axonal and dendritic processes during synapse formation [23]. Electrophysiological recordings at the glutamatergic neuromuscular junction of *Drosophila* with an extra copy of Dscam1 showed smaller and more frequent miniature EJP [24], suggesting that altered Dscam expression levels may cause impairment of synaptic transmission. In *Aplysia*, Dscam mediates the remodeling of glutamate Rs during de novo and learning-related synaptogenesis [25]. A mouse line with a spontaneous mutation in *Dscam* (*Dscam*^del17^) showed gross morphological changes in brain size and shape [22], as well as a decrease in the thickness of cortical layers and altered dendritic morphology in pyramidal neurons [22]. These mice also exhibit severe hydrocephalus, decreased motor function, and impaired motor learning ability [26]. However, it is unclear whether ASD phenotypes, such as social deficits are replicated in mice deficient for *Dscam*, and how *DSCAM* mutations lead to ASD-like cellular phenotypes at the synapse in human-iPSC-derived neuronal models.

In this study, we generated forebrain-like induced neuronal (iN) cells from the iPSCs of an ASD patient with a de novo heterozygous point mutation in the *DSCAM* gene, predicting a truncated protein expression. We investigated global gene expression, cellular phenotypes, and electrophysiological properties of the iN cells. To determine the role of DSCAM in ASD pathophysiology in vivo, we examined behavioral phenotypes, such as social interactions and repetitive behaviors in Nestin-*Dscam*^+/−^ mice.

## Materials and Methods

### Patient description

The proband, a 12-year-old boy, was born at 39 weeks’ gestation by spontaneous vaginal delivery with a birth weight of 3.5 kg after an uncomplicated pregnancy. He manifested autistic features before 36 months of age. His Autism Diagnostic Observation Schedule-2 (ADOS-2) testing demonstrated a total score of 15 (social affect score, 14; restricted and repetitive behavior score, 1) well within the range of an autism diagnosis at age five. His Autism Diagnostic Interview-Revised (ADI-R) scores were also above the autism diagnostic cutoffs. In addition, he demonstrated severe sleep problems and a significantly high level of anxiety, with crying and screaming both at school and at home, particularly with his mother. He has intellectual disability and obesity at a weight of 79 kg and height of 165 cm. He has received early intervention since the age of 3 years old, but his behavior problems have continued throughout his childhood.

### Generation of iPSCs and neuronal induction from iPSCs

Human-iPSC lines were generated from skin fibroblasts using an integration-free method, as previously described [27]. The passage numbers of the control and ASD iPSC lines used in this study were between 40 and 80. iN cells were generated as described previously [28], with minor modifications. Detailed protocols are described in the Supplementary Information.

### RNA sequencing analysis

Total RNA was extracted from the iPSC-derived control and ASD iN cells. The quality and integrity of the extracted total RNA were assessed using BioAnalyzer and the standard Illumina sequencing system protocol (TruSeq RNA sample preparation kit v2) was used to make libraries for RNA sequencing. About 300-bp-long fragments were isolated using gel electrophoresis; they were then amplified by PCR and sequenced on Illumina HiSeq X platform in the paired-end sequencing mode (2 × 150 bp reads). Bioinformatic analysis was performed as described previously [29]. Briefly, raw sequencing reads were aligned to the human genome and differential gene expression analysis was conducted using the DESeq2 method [30] and genes with at least 1.5-fold changes between groups at a false discovery rate of 5% were defined as differentially expressed genes. The Metascape tool [31] was used for functional annotations for the differentially expressed genes identified.

### Quantitative real-time PCR

Total mRNA was extracted and cDNA was synthesized as previously described [32]. To analyze gene expression changes between control and ASD iPSCs or iN cells, quantitative RT-PCR was performed using specific primers, which are presented in Supplementary Table 3.

### Knockdown experiments

For knockdown experiments, pSuper-dTomato or pSuper-H1-hDSCAM shRNA-dTomato were transfected in 5–6-week-old control iN cells and fixed with 4% paraformaldehyde 48 h after the transfection. Regarding rescue experiments, pSuper-dTomato or pSuper-H1-hDSCAM shRNA-dTomato constructs together with pcDNA3-hDSCAM(R)-FLAG construct were co-transfected into 5–6-week-old control iN cells (pSuper-dTomato: 2.5 μg, pSuper-H1-hDSCAM-shRNA-dTomato: 2.5 μg, pcDNA3-hDSCAM(R)-FLAG: 5 μg, Lipofectamine 2000 or 3000: 4–6 μl per well of 24-well plate) and the transfection mixture was then replaced with culture media 6 h after the transfection. After a 48-h incubation, the iN cells were fixed with 4% paraformaldehyde for immunocytochemical analysis.

### Immunocytochemistry

Standard immunocytochemistry was performed as described previously [33, 34]. Detailed experimental procedures with antibody information are described in the Supplementary Information.

### Proximity ligation assay (PLA)

Generic in situ PLA was performed using a Duolink kit (Sigma-Aldrich, DUO92101) according to the manufacturer’s instructions with minor changes. Detailed protocols are given in the Supplementary Information. PLA reactions were followed by immunocytochemistry with the addition of primary antibodies (PSD-95, NeuroMab, 75-028) and then fluorophore-conjugated secondary antibodies.

### Western blot analysis

Western blot analysis was performed as previously described [35]. Further details are provided in the Supplementary Information.

### Electrophysiology

Whole-cell patch-clamp recordings from iN cells and the anterior cingulate cortex (ACC) layer 2/3 cortical neurons from mouse brain slices were performed as previously described [36]. More details are provided in the Supplementary Information.

### Generation of Nestin-*Dscam*^+/−^ mice

Nestin-*Dscam*^+/^^−^ mice were produced by crossing Nestin-Cre mice with *Dscam* floxed exon 1 mice (B6.129P2-*Dscam*^*tm1.1Kzy*^, RIKEN BioResource Research Center) [37]. Mice were bred in a room with a 12–12 h light-dark cycle, and food and water were provided *ad libitum*. All behavioral experiments were conducted during the light phase and were approved by the Institutional Animal Care and Use Committees at Seoul National University (SNU-200109-2).

### Behavioral analyses

Behavioral analyses were performed as previously described [38–40] and are provided in detail in the Supplementary Information.

### Statistical analysis

We did not use any statistical methods to pre-determine the sample sizes, but our sample sizes were similar to those reported in previously published papers [28, 41]. All data were analyzed by Prism 8 software (GraphPad). Either unpaired *t*-test or Mann–Whitney test after normality tests was conducted to compare two different groups. One-way ANOVA or Kruskal–Wallis test were used to compare the means of more than two groups. Two-way (repeated measures) ANOVA was used to analyze the mean differences between groups. In all statistical analyses, significance was shown as *p < 0.05, **p < 0.01, ***p < 0.001, and ****p < 0.0001. Data are presented as the mean ± SEM.

## Results

### Clinical features and genetic information of the ASD patient

**Fig. 1:**
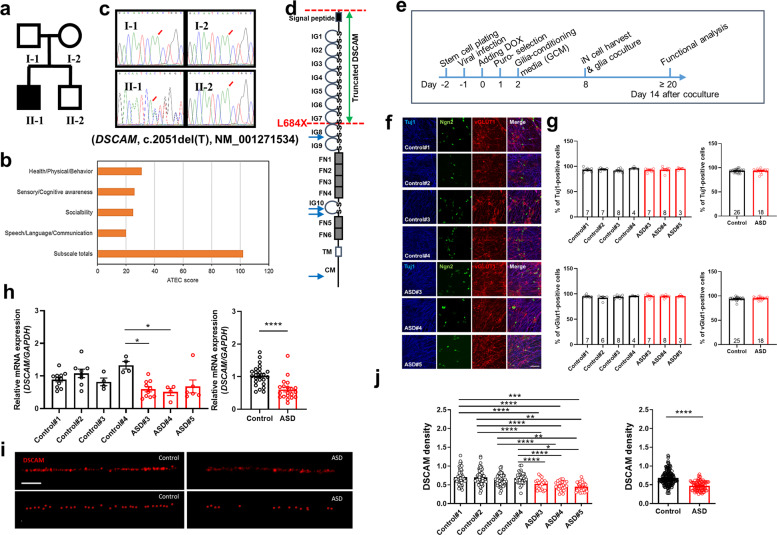
De novo single nucleotide deletion in the *DSCAM* gene of an ASD patient and generation and characterization of ASD iN cells. **a** Pedigree of the ASD family. The black-filled symbol represents the ASD patient (II-1). **b** Autism Treatment Evaluation Checklist (ATEC) score of the ASD patient before intervention. **c** Whole exome sequencing was performed on four family members (I-1, I-2, II-1, and II-2). Confirmation of the identified single nucleotide deletion in the *DSCAM* gene. The single nucleotide deletion (*DSCAM*, c.2051del) identified in the ASD patient was validated by Sanger sequencing of the genomic DNA from four family members. The *DSCAM* transcript, NM_001271534 (RefSeq sequence) with the single nucleotide deletion (c.2051del; cDNA position 2051) is translated into a truncated form of the DSCAM protein due to an early stop codon at amino acid position 684, leucine (pL684X). **d** Schematic diagram of the DSCAM protein with the termination site (L684X), which is located between the immunoglobulin (IG) 7 and 8 domains of DSCAM. Other predicted truncation mutations reported previously (Satterstrom et al. [62]) are indicated by blue arrows (IG immunoglobulin domain, FN fibronectin type III domain, TM transmembrane domain, CM cytoplasmic domain). **e** Timetable of the differentiation procedure of iN cells from iPSCs. **f**, **g** Immunocytochemical analysis of the differentiation of iPSCs to iN cells using Tuj1 (early neuronal marker) and vGlut1 (glutamatergic neuronal marker) antibodies revealed no difference between ASD and control iN cells. The number in the bars represents the independent culture number. (Kruskal–Wallis test for upper and lower left panels, ns, not significant, unpaired *t*-test for upper right panel, ns, not significant, Mann–Whitney test for lower right panel, ns, not significant). Scale bar: 50 μm. **h** *DSCAM* mRNA levels were significantly decreased in ASD iN cells [Control: *n* = 27, ASD: *n* = 20 (Control#1: *n* = 11, Control#2: *n* = 8, Control#3: *n* = 4, Control#4: *n* = 4, ASD#3: *n* = 10, ASD#4: *n* = 4, ASD#5: *n* = 6), Kruskal–Wallis test for left panel (*p* < 0.01), followed by Dunn’s multiple comparisons test (Control#4 vs. ASD#3, *p < 0.05, Control#4 vs. ASD#4, *p < 0.05), unpaired *t*-test for right panel (****p < 0.0001)]. **i**, **j** Immunocytochemical analysis of DSCAM expression in iN cells. DSCAM expression (the number of red signals) in the neurites (**i**) was significantly reduced in ASD iN cells as compared to control iN cells (**j**) [one-way ANOVA for left panel (interaction, *p* < 0.0001, *F*_(6, 227)_ = 16.47) followed by Tukey’s multiple comparisons test (Control#1 vs. ASD#3, ***p < 0.001, Control#1 vs. ASD#4, ****p < 0.0001, Control#1 vs. ASD#5, ****p < 0.0001, Control#2 vs. ASD#3, **p < 0.01, Control#2 vs. ASD#4, ****p < 0.0001, Control#2 vs. ASD#5, ****p < 0.0001, Control#3 vs. ASD#4, **p < 0.01, Control#3 vs. ASD#5, ****p < 0.0001, Control#4 vs. ASD#3, *p < 0.05, Control#4 vs. ASD#4, ****p < 0.0001, Control#4 vs. ASD#5, ****p < 0.0001), Mann–Whitney test for right panel, ****p < 0.0001]. Scale bar:100 μm.

The patient (Fig. 1a, black-filled square in the pedigree), a 12-year-old boy, presented the clinical signs of ASD, which emerged at age 3. Subscale totals in the Autism Treatment Evaluation Checklist before medical intervention were above 102, which indicates very severe autism in the 90th percentile (Fig. 1b). Patients with scores less than 50 are within the 30th percentile and are considered to have a good chance of being semi-independent. The patient’s adaptation to nursery school was difficult. His mother described his behavioral disorders, which included incessant restlessness and hyperactivity, an inability to listen, over-eating, substantial sleeping and communication disorders, and a lack of speech. To identify genetic mutations in the patient, we performed whole exome sequencing of his four family members and found a single* de novo* mutation in the *DSCAM* gene only in the patient, identified as a single c.2051del (cDNA position 2051) by Sanger sequencing (Fig. 1c). We predicted that the deletion would generate a truncated form of the DSCAM protein due to the introduction of a premature termination codon (L684X) located between immunoglobulin domains 7 and 8 (Fig. 1d).

### Generation and characterization of ASD induced neuronal (iN) cells

To investigate the pathophysiological effect of the novel premature termination mutation in the *DSCAM* gene, patient-specific ASD iPSC lines (ASD#3, ASD#4, and ASD#5) and control iPSC lines (Control#1, Control#2, and Control#3) were generated from skin fibroblasts of the ASD patient and healthy controls (Supplementary Table 1). We also used another control iPSC line (Control#4) which was generated and characterized in a previous study [42]. The ASD and control iPSC colonies showed a typical round morphology, with small, tightly packed cells (Supplementary Fig. 1a). Pluripotency of each iPSC line was validated by alkaline phosphatase activity, immunocytochemistry, and RT-PCR analyses using stem cell markers (Oct3/4, Sox2, Rex1, and Nanog) and pluripotency markers (Oct3/4, SSEA4, Tra1-60, and Tra1-81), respectively (Supplementary Fig. 1a–d, Supplementary Table 2). Each control or ASD iPSC line displayed a normal karyotype (Supplementary Fig. 2a). All control iPSC (#1, 2, 3, 4) and ASD iPSC (#3, 4, 5) lines tested negative for mycoplasma (Supplementary Fig. 2b).

To examine cellular phenotypes caused by the *DSCAM* mutation, forebrain-like iN cells were generated from the above iPSCs using previously described protocols [28] with minor modifications (Fig. 1e). The *DSCAM* mutation was confirmed in each step of the process of fibroblasts to ASD iPSCs to ASD iPSCs-derived iN cells (Supplementary Fig. 2c, Supplementary Table 2). The relative neural differentiation ratio from iPSCs to iN cells was measured by counting Tuj1-immunopositive cells vs. Neurogenin2-expressing (GFP-positive) cells at 2 weeks of glia co-culture, with no difference in the neural differentiation rate between ASD and control iN cells (Fig. 1f, g). We also performed vGlut1 immunostaining with 2-week-old iN cell cultures to check the efficiency of Ngn2-induction and >95% of iN cells expressed vGlut1 (Fig. 1f, g), suggesting that pure glutamatergic iN cells were induced by Ngn2 expression, consistent with a previous report [28]. Next, we examined whether the heterozygous *DSCAM* mutation affected the DSCAM expression level of ASD iN cells. The mRNA expression of *DSCAM* was significantly reduced in ASD iN cells as compared to controls (Fig. 1h). The significantly lower expression of the DSCAM protein was detected by immunocytochemistry in the dendrites of ASD iN cells (Fig. 1i, j) as compared to controls, indicating that the single c.2051del (cDNA position 2051) may affect the expression level of DSCAM (Fig. 1h–j).

We next examined axonal or dendritic development in control or ASD iN cells. Thus, we investigated axonal growth in iN cells at day 3 after glia co-culture by visualizing axons with tubulin immunostaining, which lacks a MAP2 signal (Supplementary Fig. 3a). Axonal length was significantly decreased in ASD iN cells as compared to control iN cells (Supplementary Fig. 3b). We also examined dendritic growth at day 6 after glia co-culture. Dendritic complexity as measured by Sholl analyses was unchanged in ASD iN cells as compared to control iN cells (Supplementary Fig. 3c–e). To examine spinogenesis, we examined spines by PSD-95 immunostaining in 5–6-week-old iN cell cultures and observed comparable spine density in both control and ASD iN cells (Supplementary Fig. 6d).

### RNA sequencing analysis

**Fig. 2:**
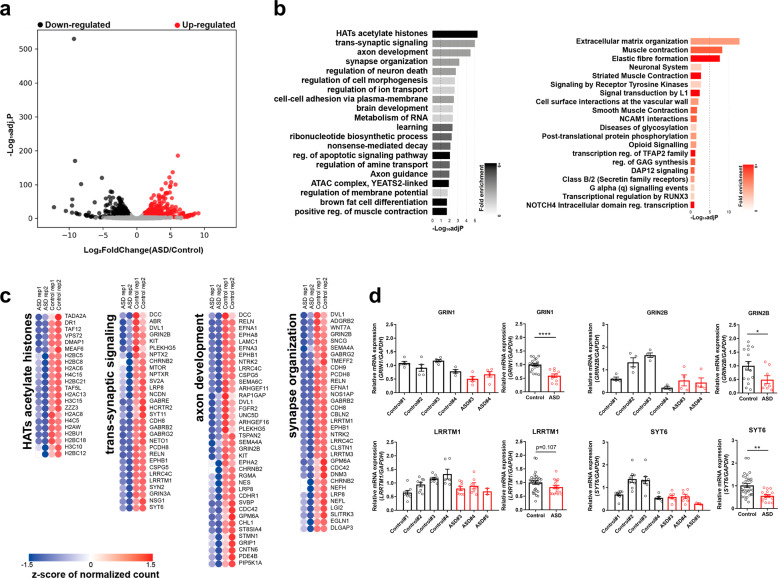
Transcriptomic analysis of ASD iN cells. **a** A volcano plot for differentially expressed genes between ASD and control iN cells. Significantly upregulated genes are represented by red dots and black dots indicate significantly downregulated genes in ASD iN cells. The X-axis is the log2-transformed gene expression in ASD iN cells divided by that in control iN cells. The Y-axis represents the *p* value (−log10) adjusted by the multiple testing correction. **b** Gene ontology term and functional pathway enrichments in upregulated (red) genes and downregulated (black) genes in ASD iN cells by Metascape. The adjusted *p* values of significantly enriched terms or pathways are shown as bar plots (−log10adjP). **c** Representative heatmaps for the top four enriched terms or pathways (HATs acetylate histones, trans-synaptic signaling, axon development, and synapse organization) for the down downregulated genes in ASD iN cells. **d** Quantitative RT-PCR analyses of mRNA expression level of *NR1*, *NR2B*, *LRRTM1*, and *SYT6* in 3-week-old iN cells. Each gene expression was normalized to *GAPDH* expression. The NMDA-R components, *NR1* and *NR2B*, are decreased in ASD iN cells. For NR1 [Control: *n* = 19, ASD: *n* = 10 (Control#1: *n* = 5, Control#2: *n* = 5, Control#3: *n* = 5, Control#4: *n* = 4, ASD#3: *n* = 5, ASD#4: *n* = 5), unpaired *t*-test for right panels (****p < 0.0001)]. For NR2B [Control: *n* = 15, ASD: *n* = 8 (control#1: *n* = 4, control#2: *n* = 4, control#3: *n* = 4, control#4: *n* = 3, ASD#3: *n* = 4, ASD#4: *n* = 4), unpaired *t*-test for right panel, *p < 0.05]. For LRRTM1 [Control: *n* = 30, ASD: *n* = 17 (Control#1: *n* = 8, Control#2: *n* = 8, Control#3: *n* = 8, Control#4: *n* = 6, ASD#3: *n* = 8, ASD#4: *n* = 7, ASD#5: *n* = 2), unpaired *t*-test for right panels, not significant (*p* = 0.107)]. For SYT6 [Control: *n* = 30, ASD: *n* = 16 (Control#1: *n* = 8, Control#2: *n* = 8, Control#3: *n* = 8, Control#4: *n* = 6, ASD#3: *n* = 7, ASD#4: *n* = 7, ASD#5: *n* = 2), unpaired *t*-test for right panel (**p < 0.01)].

To delineate the molecular pathology mechanisms for the identified de novo variants, we performed RNA sequencing analysis and compared the transcriptomic profiles between ASD iN cells (#3) and control iN cells (#1 and #2). A total of 1805 differentially expressed genes (upregulated, 877 and downregulated, 928) were identified (Fig. 2a) and functional annotations analysis using known pathways and gene ontology was conducted to investigate the enriched molecular signatures in the differentially expressed genes. Of note, pathways enriched in downregulated genes in ASD iN cells are related to neuronal processes, such as “trans-synaptic signaling”, “axon development”, “synapse organization”, and “regulation of neuron death” (Fig. 2b, left panel and Fig. 2c). In contrast, pathways or terms related to general developmental processes, such as “extracellular matrix organization” and “elastic fiber formation” are enriched in the upregulated genes in ASD iN cells (Fig. 2b, right panel). Interestingly, we found that the expression of NMDA-R subunits, such as *NR1*, *NR2B*, and *NR3A* were reduced in ASD iN cells as compared to control iN cells.

We performed quantitative RT-PCR to validate the RNA sequencing analysis. Among several synapse-related genes, the components of NMDA-R, *NR1*, and *NR2B*, are decreased in ASD iN cells (Fig. 2d, upper panel). Specifically, *NR1* showed a more severe decrease than *NR2B*. Furthermore, the genes related to trans-synaptic signaling, *LRRTM1* and *SYT6*, are also decreased in ASD iN cells as compared to control iN cells (Fig. 2d, bottom panel).

### Compromised NMDA currents in ASD iN cells are rescued by exogenous expression of full-length DSCAM

**Fig. 3:**
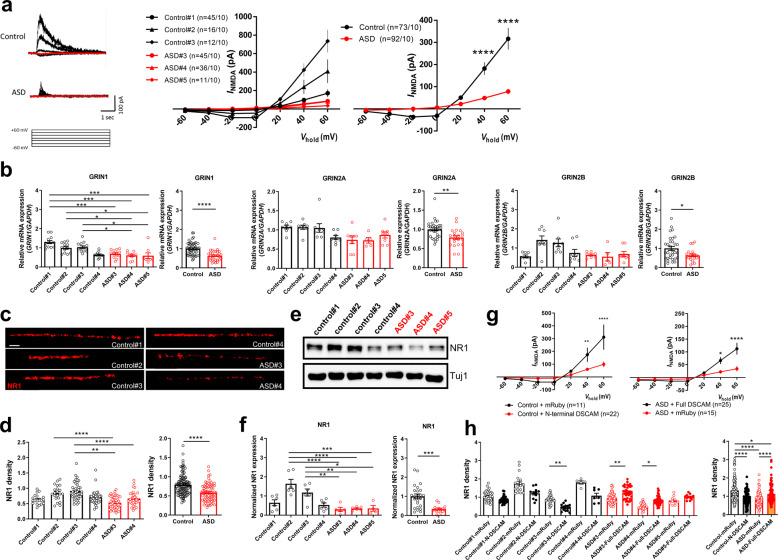
NMDA-R-mediated currents are compromised in ASD iN cells. **a** Voltage dependency of NMDA-R-mediated currents (*I*_*NMDA*_) in control and ASD iN cells. Note the significantly lower *I*_*NMDA*_ at +40 and +60 mV in ASD iN cells (Control: *n* = 73, ASD: *n* = 92 from ten independent cultures; two-way repeated-measures ANOVA, interaction, *F*_(6, 228)_ = 6.200, *p* < 0.0001; Bonferroni post hoc test, +40 mV, ****p < 0.0001; +60 mV, ****p < 0.0001). **b** Quantitative RT-PCR analysis of normalized *NR1*/*NR2A*/*NR2B* gene expression in control and ASD iN cells. The expression of *NR1, NR2A*, and *NR2B* was significantly reduced in ASD iN cells. For NR1 [Control: *n* = 51, ASD: *n* = 31 (Control#1: *n* = 13, Control#2: *n* = 13, Control#3: *n* = 13, Control#4: *n* = 12, ASD#3: *n* = 13, ASD#4: *n* = 10, ASD#5: *n* = 8), Kruskal–Wallis test for left panels (*p* < 0.0001) followed by Dunn’s multiple comparisons test (Control#1 vs. Control#4, ****p < 0.0001, Control#1 vs. ASD#3, ***p < 0.001, Control#1 vs. ASD#4, ***p < 0.001, Control#1 vs. ASD#5, ***p < 0.001, Control#2 vs. Control#4, *p < 0.05, Control#2 vs. ASD#4, *p < 0.05, Control#2 vs. ASD#5, *p < 0.05, Control#3 vs. Control#4, *p < 0.05, Control#3 vs. ASD#4, *p < 0.05, Control#3 vs. ASD#5, *p < 0.05), unpaired *t*-test for right panels (****p < 0.0001)]. For NR2A [Control: *n* = 32, ASD: *n* = 22 (Control#1: *n* = 8, Control#2: *n* = 8, Control#3: *n* = 8, Control#4: *n* = 8, ASD#3: *n* = 8, ASD#4: *n* = 6, ASD#5: *n* = 8), Kruskal–Wallis test for left panels (*p* < 0.01) followed by Dunn’s multiple comparisons test (ns, not significant), unpaired *t*-test for right panels (**p < 0.01)]. For NR2B [Control: *n* = 32, ASD: *n* = 21 (Control#1: *n* = 8, Control#2: *n* = 8, Control#3: *n* = 8, Control#4: *n* = 8, ASD#3: *n* = 8, ASD#4: *n* = 5, ASD#5: *n* = 8), Kruskal–Wallis test for left panels (*p* < 0.01) followed by Dunn’s multiple comparisons test (ns, not significant), unpaired *t*-test for right panels (*p < 0.05)]. **c** Immunocytochemical analysis of NR1 expression in control and ASD iN cells. Scale bar: 50 μm. **d** The expression of NR1 was significantly reduced in ASD iN cells [Control: *n* = 51, ASD: *n* = 31 (Control#1: *n* = 13, Control#2: *n* = 13, Control#3: *n* = 13, Control#4: *n* = 12, ASD#3: *n* = 13, ASD#4: *n* = 10, ASD#5: *n* = 8), Kruskal–Wallis test for left panel (*p* < 0.0001) followed by Dunn’s multiple comparisons test (Control#1 vs. Control#3, **p < 0.01, Control#2 vs. ASD#3, ****p < 0.0001, Control#3 vs. Control#4, *p < 0.05, Control#3 vs. ASD#3, ****p < 0.0001, Control#3 vs. ASD#4, **p < 0.01), Mann–Whitney test for right panel (****p < 0.0001)]. **e**, **f** Western blot analysis of NR1 expression from control and ASD iN cells. **e** Representative Western blot images of NR1 expression. **f** Quantitative analysis of normalized NR1 expression in control and ASD iN cells. Note that the NR1 expression was significantly reduced in ASD iN cells as compared to control iN cells [Control: *n* = 24, ASD: *n* = 15 (Control#1: *n* = 6, Control#2: *n* = 6, Control#3: *n* = 6, Control#4: *n* = 6, ASD#3: *n* = 5, ASD#4: *n* = 6, ASD#5: *n* = 4), one-way ANOVA for left panel (*p* < 0.0001, *F*_(6,32)_ = 10.99) followed by Tukey’s multiple comparisons test (Control#1 vs. Control#2, ***p < 0.001, Control#2 vs. Control#4, ***p < 0.001, Control#2 vs. ASD#3, ****p < 0.0001, Control#2 vs. ASD#4, ****p < 0.0001, Control#2 vs. ASD#5, ***p < 0.001, Control#3 vs. ASD#3, **p < 0.01, Control#3 vs. ASD#4, **p < 0.01, Control#3 vs. ASD#5, *p < 0.05), unpaired *t*-test for right panel (***p < 0.001)]. **g** Exogenous N-terminal DSCAM expression in control iN cells led to significantly decreased *I*_*NMDA*_ at +40 and +60 mV (Control + mRuby: *n* = 11, Control + N-terminal DSCAM: *n* = 22; two-way repeated-measures ANOVA, interaction, *F*_(6, 186)_ = 7.892, *p* < 0.0001; Bonferroni post hoc test, +40 mV, *p* = 0.0054; +60 mV, *p* < 0.0001). Exogenous overexpression of human WT DSCAM in ASD iN cells significantly increased *I*_*NMDA*_ at +40 and +60 mV (ASD + Full DSCAM: *n* = 25, ASD + mRuby: *n* = 15; two-way repeated-measures ANOVA, interaction, *F*_(6, 228)_ = 6.200, *p* < 0.0001; Bonferroni post hoc test, +40 mV, *p* < 0.05; +60 mV, *p* < 0.0001). **h** Exogenous N-terminal DSCAM expression in control iN cells led to significantly decreased NR1 density (Control + mRuby: *n* = 86; Control#1 + mRuby: *n* = 37, Control#2 + mRuby: *n* = 17, Control#3 + mRuby: n = 27, Control#4 + mRuby: n = 5, Control + N-terminal DSCAM: n = 65; Control#1 + N-terminal DSCAM: n = 31, Control#2 + N-terminal DSCAM: *n* = 11, Control#3 + N-terminal DSCAM: *n* = 16, Control#4 + N-terminal DSCAM: *n* = 7) and exogenous overexpression of WT full-length DSCAM in ASD iN cells significantly increased NR1 expression (ASD + mRuby: *n* = 112; ASD#3 + mRuby: *n* = 69, ASD#4 + mRuby: *n* = 33, ASD#5 + mRuby: *n* = 10, ASD + Full-length DSCAM: *n* = 93; ASD#3 + Full-length DSCAM: *n* = 36, ASD#4 + Full-length DSCAM: *n* = 45, ASD#5 + Full-length DSCAM: *n* = 12). Kruskal–Wallis test for left panel (*p* < 0.0001) followed by Dunn’s multiple comparisons test (Control#3 + mRuby vs. Control#3 + N-terminal DSCAM, **p < 0.01, ASD#3 + mRuby vs. ASD#3 + N-terminal DSCAM, *p < 0.05, ASD#4 + mRuby vs. ASD#4 + N-terminal DSCAM, **p < 0.01), Kruskal–Wallis test for right panel (*p* < 0.0001) followed by Dunn’s multiple comparisons test (Control + mRuby vs. Control + N-terminal DSCAM, ****p < 0.0001, Control + mRuby vs. ASD + mRuby, ****p < 0.0001, Control + mRuby vs. ASD + Full-length DSCAM, *p < 0.05, ASD + mRuby vs. ASD + Full-length DSCAM, ****p < 0.0001).

As our transcriptome analyses revealed the reduced expression of NMDA-R subunits and NMDA-R dysfunctions have been implicated in the pathogenesis of ASD, we investigated whether NMDA-R-mediated currents are altered in ASD iN cells. Interestingly, the amplitude of NMDA-R currents (*I*_*NMDA*_) was significantly decreased in ASD iN cells compared to control iN cells (Fig. 3a). Consistently, the mRNA levels of NMDA-R components (*NR1*, *NR2A* and *NR2B*) were significantly lower in ASD iN cells compared to control iN cells (Fig. 3b). Specifically, *NR1* shows severe decrease in ASD iN cells, and we focused on NR1 for followed experiments. The protein levels of NR1, as detected by immunocytochemistry (Fig. 3c, d) and immunoblotting (Fig. 3e, f), were also reduced in ASD iN cells.

In our next experiment, we examined the basic electrical properties of control and ASD iN cells. The resting membrane potential of ASD iN cells was approximately −45 mV at 2 weeks after glia co-culture, similar to that of control iN cells (Supplementary Fig. 4a). Membrane capacitance (Supplementary Fig. 4b) also did not differ between ASD and control iN cells. However, input resistance was significantly decreased in ASD iN cells (Supplementary Fig. 4c). We observed an increase in neuronal excitability in ASD iN cells, with a larger amplitude and shorter half-width of the action potential (Supplementary Fig. 4d–g). In addition, we examined whether the heterozygous *DSCAM* mutation affected any synaptic properties of ASD iN cells. We analyzed spontaneous synaptic activity by measuring miniature EPSC, with no changes in frequency but with a slight increase in the amplitude of mEPSC in ASD iN cells (Supplementary Fig. 4h, i).

In order to determine whether the truncated form of DSCAM (might be generated by the genetic mutation) affected NMDA-R currents (*I*_*NMDA*_), we examined NMDA-R current changes upon the expression of either the full-length or truncated form of DSCAM in ASD and control iN cells, respectively. When the truncated form of DSCAM (N-terminal DSCAM) was transfected in control iN cells, a significant decrease in *I*_*NMDA*_ at the +40 and +60 mV holding potentials was observed (Fig. 3g, left panel). In contrast, when the full-length DSCAM was expressed in ASD iN cells, amplitude of *I*_*NMDA*_ at the +40 and +60 mV holding potentials was significantly increased (Fig. 3g, right panel). Expression of NR1 detected by immunocytochemistry was decreased when the truncated form of DSCAM was introduced in control iN cells (Fig. 3h) while it was increased when the full-length DSCAM was expressed in ASD iN cells (Fig. 3h). In addition, when endogenous DSCAM was knocked down by a specific shRNA (Supplementary Fig. 5a), NR1 and DSCAM expression were significantly decreased in the control iN cells (Supplementary Fig. 5b–e). The decreases of both DSCAM and NR1 density were rescued to control levels by the reintroduction of shRNA-resistant DSCAM [DSCAM(R)] in control iN cells in which endogenous DSCAM was knocked down by shRNA (Supplementary Fig. 5f–i). Consequently, these data suggest that wild-type (WT) DSCAM is essential for proper NR1 expression and normal NMDA-R currents.

### Co-localization of DSCAM with NR1 in dendritic spines and pERK1/2 activity is significantly reduced in ASD iN cells

**Fig. 4:**
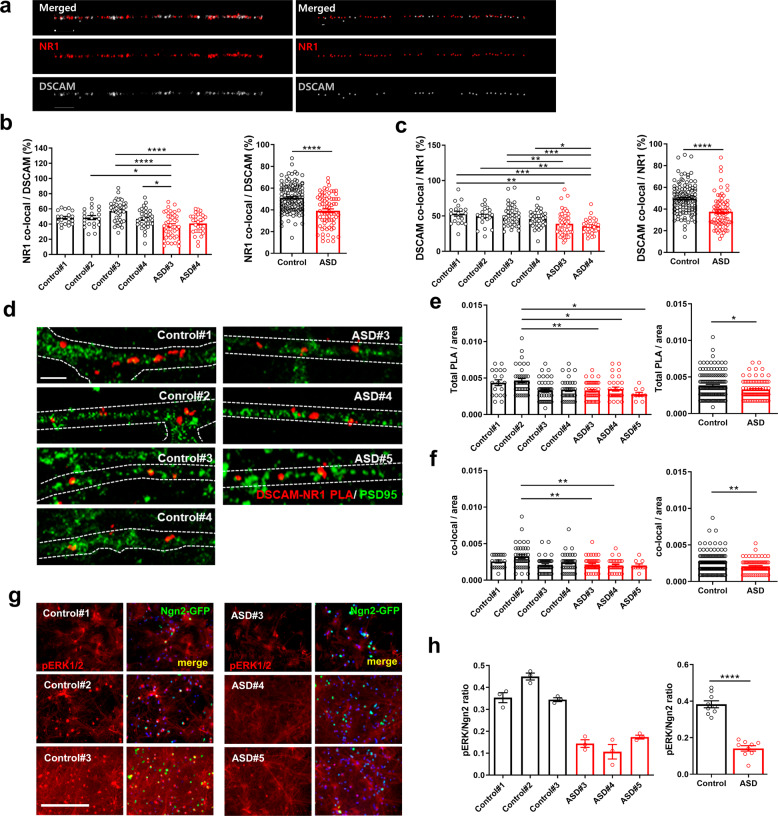
Reduced co-localization of DSCAM with NR1 and phospho-ERK level in ASD iN cells. **a** Immunocytochemical and processed by IMARIS program images of NR1 and DSCAM co-localization in control and ASD iN cells. Scale bar: 5 μm. Quantitative analysis of percent co-localization of NR1 over DSCAM [Control: *n* = 117, ASD: *n* = 74 (Control#1: *n* = 19, Control#2: *n* = 21, Control#3: *n* = 41, Control#4: *n* = 36, ASD#3: *n* = 45, ASD#4: *n* = 29), one-way ANOVA for left panel (interaction, *p* < 0.0001, *F*_(5, 185)_ = 10.18) followed by Bonferroni post hoc test (Control#2 vs. ASD#3, *p < 0.05, Control#3 vs. ASD#3, ****p < 0.0001, Control#3 vs. ASD#4, ****p < 0.0001, Control#4 vs. ASD#4, *p < 0.05), unpaired *t*-test for right panel, ****p < 0.0001] (**b**) or DSCAM over NR1 [Control: *n* = 117, ASD: *n* = 74 (Control#1: *n* = 19, Control#2: *n* = 21, Control#3: *n* = 41, Control#4: *n* = 36, ASD#3: *n* = 45, ASD#4: *n* = 29), one-way ANOVA for left panel (interaction, *p* < 0.0001, *F*_(5, 185)_ = 7.457) followed by Bonferroni post hoc test (Control#1 vs. ASD#3, **p < 0.01, Control#1 vs. ASD#4, ***p < 0.001, Control#2 vs. ASD#4, **p < 0.01, Control#3 vs. ASD#3, **p < 0.01, Control#3 vs. ASD#4, ***p < 0.001, Control#4 vs. ASD#4, *p < 0.05), unpaired *t*-test for right panel, **** *p* < 0.0001] (**c**) in control and ASD iN cells. Note that co-localization of DSCAM and NR1 is significantly reduced in ASD iN cells as compared to control iN cells. **d** Proximity ligation assay (PLA) of the co-localization between NR1 and DSCAM in iN cells. Note that the PLA signals (red puncta) were clearly observed between DSCAM and NR1. Scale bar: 100 μm. **e** Quantitative analysis of normalized total PLA signal resulted in a significant decrease in ASD iN cells [Control: *n* = 141, ASD: *n* = 85 (Control#1: *n* = 19, Control#2: *n* = 41, Control#3: *n* = 46, Control#4: *n* = 35, ASD#3: *n* = 44, ASD#4: *n* = 31, ASD#5: *n* = 10), Kruskal–Wallis test for left panel (*p* < 0.0001) followed by Dunn’s multiple comparison test (Control#2 vs. Control#3, **p < 0.01, Control#2 vs. ASD#4, *p < 0.05, Control#2 vs. ASD#5, *p < 0.05), Mann–Whitney test for right panel (*p < 0.05)]. Quantification of total PLA was calculated as the number of total PLA divided by image area (µm^2^). **f** Co-localization between PLA signals and PSD-95 puncta also decreased in ASD iN cells [Control: *n* = 141, ASD: *n* = 85 (Control#1: *n* = 19, Control#2: *n* = 41, Control#3: *n* = 46, Control#4: *n* = 35, ASD#3: *n* = 44, ASD#4: *n* = 31, ASD#5: *n* = 10), Kruskal–Wallis test for left panel (*p* < 0.001) followed by Dunn’s multiple comparison test (Control#2 vs. ASD#3, **p < 0.01, Control#2 vs. ASD#4, **p < 0.01), Mann–Whitney test for right panel (**p < 0.01)]. Quantification of co-localization of PLA signals and PSD-95 puncta was evaluated as the number of co-localized puncta divided by the image area (μm^2^). **g** Representative immunocytochemical images of phospho-ERK1/2 staining in iN cells. Scale bar: 200 μm. **h** Quantification of immunocytochemical analysis of phospho-ERK1/2 staining showed a significant decrease of phospho-ERK1/2-positive cells in ASD iN cell cultures as compared to control iN cell cultures (Control: *n* = 9, ASD: *n* = 9 from three independent cultures for each line, unpaired *t*-test for right panel, ****p < 0.0001).

To define how DSCAM affected NMDA-R currents, we examined the co-localization of DSCAM with an NMDA-R subunit, NR1, in iN cells. Immunoreactive (IR) signals for NR1 and DSCAM were widely distributed throughout the cell bodies and dendrites and showed about 50% co-localization in dendrites (Fig. 4a–c, Supplementary Fig. 6). This co-localization between DSCAM and NR1 was significantly reduced in dendrites of ASD iN cells (Fig. 4a–c). To further confirm a direct interaction between NR1 and DSCAM, we performed an in situ PLA in 6-week-old control and ASD iN cells. First, we applied in situ PLA to visualize the interaction of DSCAM with NR1, followed by immunostaining with anti-PSD-95 antibodies. A considerable amount of PLA signals was present, indicating the interaction between DSCAM and NR1 in control and ASD iN cells (Fig. 4d). However, it was significantly reduced in ASD iN cells (Fig. 4e, Supplementary Fig. 7a), consistent with reduced co-localization of IR signals for DSCAM and NR1 (Fig. 4a–c). However, no PLA signal was found in a control PLA experiment using DSCAM antibody alone (data not shown). In addition, a significant percentage of the DSCAM-NR1 PLA signal (~70%) was co-localized with PSD-95 IR signals (Fig. 4d, f, Supplementary Fig. 7b–d), suggesting that DSCAM interacts with NMDA-Rs in dendritic spines and plays a role in modulating NMDA-R currents.

Extracellular signal-regulated kinase (ERK) activity is modulated by NMDA-R activation and is synchronized with NMDA-R currents [43]. To gain insight into the potential downstream consequences of DSCAM and NMDA-R co-localization, we measured phospho-ERK levels in ASD and control iN cells. We observed a low level of pERK IR signal (Fig. 4g) and a significant decrease in the pERK/Ngn2 ratio in ASD iN cells as compared to control iN cells (Fig. 4g, h). These results raise the possibility that DSCAM interacts with NMDA-Rs in the spine to regulate the downstream intracellular ERK signaling pathway in control iN cells, but their interactions are reduced in ASD iN cells.

### Generation of Nestin-*Dscam*^+/−^ mice

Recently, variants in the *DSCAM* gene were identified in many ASD patients [16–20], and several *Dscam* KO mouse models have been established. However, autistic phenotypes are not yet reported in these mouse models. To examine the effect of *Dscam* mutation on behavioral phenotypes in vivo, we generated an ASD mouse model by crossing the Nestin-Cre mouse [44] and the floxed *Dscam* exon 1 mouse [37], which causes the *Dscam* gene to be heterozygously deleted, specifically in neural stem cells (NSCs) (Supplementary Fig. 8a). Western blot analysis on the ACC, a brain region associated with social behaviors [41, 45], showed that Dscam expression in the heterozygous Nestin-*Dscam*^+/^^−^ mice was significantly reduced as compared to WT littermates (Supplementary Fig. 8b). Notably, the body weight of Nestin-*Dscam*^+/−^ mice was significantly lower compared to WT littermates (Supplementary Fig. 8c).

To examine whether the basal behavior of Nestin-*Dscam*^+/−^ mice was altered, we used the open-field test (Supplementary Fig. 8d–f). Both WT and Nestin-*Dscam*^+/−^ mice spent similar time in the center zone (Supplementary Fig. 8d, e), which indicates no change in anxiety-like behavior in the Nestin-*Dscam*^+/^^−^ mice. There was also no significant difference in the distance moved between Nestin-*Dscam*^+/−^ and WT littermates during 30 min in the open-field test (Supplementary Fig. 8f), suggesting that locomotive activity was normal in Nestin-*Dscam*^+/−^ mice.

### Nestin-*Dscam*^+/−^ mice are impaired in social behaviors and NMDA-R functions

**Fig. 5:**
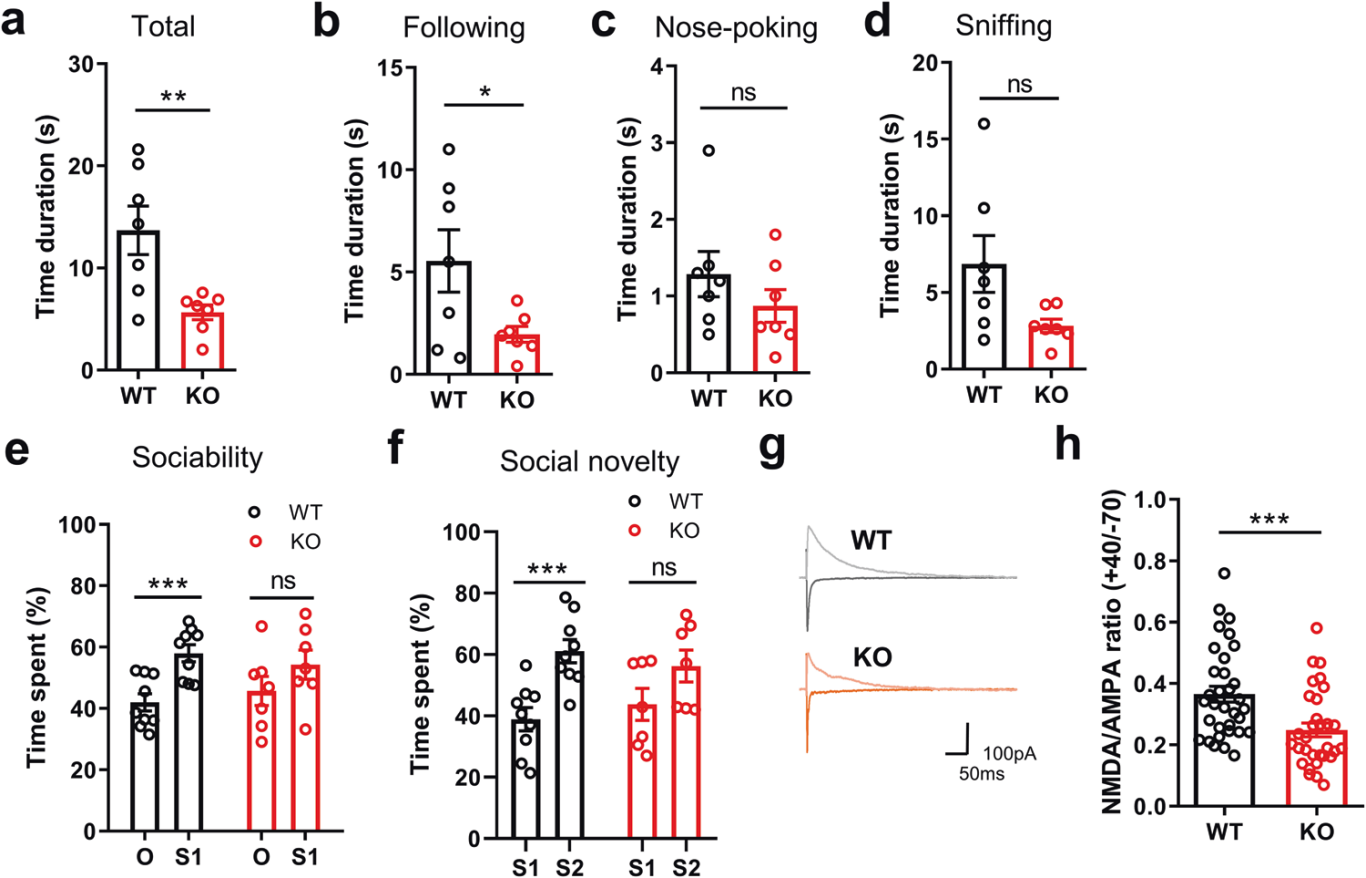
Nestin-*Dscam*^+/−^ mice exhibit impaired social behaviors. **a** The total duration of reciprocal interactions was significantly decreased in Nestin-*Dscam*^+/−^ mice as compared to WT littermates (WT: *n* = 7, KO: *n* = 7, unpaired *t*-test, **p < 0.01). **b**–**d** Duration of following, nose-poking, and sniffing behaviors in WT and Nestin-*Dscam*^+/-^ mice. Time spent on following behavior (**b**) but not nose-poking (**c**) and sniffing (**d**) behaviors was significantly decreased in Nestin-*Dscam*^+/−^ mice as compared to WT littermates (WT: *n* = 7, KO: *n* = 7, unpaired *t*-test, *p < 0.05, ns, not significant). **e**, **f** The three-chamber test was used to measure changes in sociability and social novelty in Nestin-*Dscam*^+/−^ mice. **e** Percent time in contact with the stranger 1 mouse was significantly increased as compared to the object in WT mice but not in Nestin-*Dscam*^+/−^ mice (WT: *n* = 9, KO: *n* = 7, unpaired *t*-test, ***p < 0.001, ns, not significant). **f** In the social novelty test, Nestin-*Dscam*^+/−^ mice showed no preference between the stranger 1 and stranger 2 mice, whereas WT littermates spent more time in contact with the stranger 2 as compared to stranger 1 mouse (WT: *n* = 9, KO: *n* = 7, unpaired *t*-test, ***p < 0.001, ns, not significant). **g**, **h** AMPA-R and NMDA-R currents were estimated in the presence of picrotoxin (50 µM) by varying holding potentials from −70 to +40 mV. The NMDA/AMPA ratio was significantly reduced in Nestin-*Dscam*^+/−^ mice as compared to WT littermates (WT: *n* = 33/4 animals, KO: *n* = 31/4 animals, Mann–Whitney test, ***p < 0.001).

Next, the social interaction behaviors of Nestin-*Dscam*^+/−^ mice were measured using reciprocal interaction and three-chamber tests [10, 39, 46, 47]. In the reciprocal interaction test, we observed a decreased total interaction time and following time but not nose-poking and sniffing time in Nestin-*Dscam*^+/−^ mice as compared to WT littermates (Fig. 5a–d). In the three-chamber test, WT mice spent more time with the stranger mouse (stranger 1) than with the object. In contrast, the Nestin-*Dscam*^+/^^−^ mice spent less time interacting with the stranger mouse than with the object, suggesting impaired sociability (Fig. 5e). Next, another stranger mouse (stranger 2) was placed in one chamber instead of the object, and the test mouse was allowed to explore the chamber freely. Nestin-*Dscam*^+/−^ mice showed a decreased level of interest in the stranger 2 mouse as compared to WT mice (Fig. 5f), suggesting a social novelty deficit in Nestin-*Dscam*^+/−^ mice. Social communication as measured by the ultrasonic vocalization test [48, 49] was not different in Nestin-*Dscam*^+/−^ mice as compared to WT littermates (Supplementary Fig. 9a, b). Therefore, Nestin-*Dscam*^+/^^−^ mice showed reduced sociability and social novelty as compared to WT littermates, which reflects some behavioral characteristics of ASD patients.

An increased level of repetitive behavior is also a feature of ASD [50, 51]. To examine whether the Nestin-*Dscam*^+/^^−^ mice showed any repetitive behaviors, we performed the marble burying test and monitored for various repetitive behaviors in the mouse’s home cage. Nestin-*Dscam*^+/−^ mice and WT littermates showed no performance difference in the marble-burying test (Supplementary Fig. 9c). When we analyzed the time spent on repetitive behaviors, such as self-grooming, digging, and rearing in the home cage, there was no difference between Nestin-*Dscam*^+/−^ mice and WT littermates (Supplementary Fig. 9d–f).

Finally, we examined the ratio of NMDA-R to AMPA-R-mediated synaptic currents from layer 2/3 pyramidal neurons in the ACC. Consistent with the results from the iN cells derived from our ASD patient (Fig. 3c, d, Supplementary Fig. 4), the NMDA/AMPA ratio was significantly lower in the Nestin-*Dscam*^+/^^−^ mice as compared to WT littermates (Fig. 5g, h), suggesting that NMDA-R hypofunction underlies autistic behaviors found in Nestin-*Dscam*^+/−^ mice.

## Discussion

Although numerous ASD-related genetic variations are reported [52, 53], synaptic dysfunction is suggested as a core mechanism underlying ASD pathophysiology [54, 55]. Among the synaptic proteins associated with ASD, NMDA-R has garnered much attention for its importance in synaptic transmission in the mammalian excitatory synapse [56]. In animal studies, many ASD models with mutations in various genes exhibit NMDA-R dysfunction [10–13]. Mice lacking neuroligin-1 showed reduced NMDA-R function in the hippocampus and striatum [12, 57, 58]. Shank2 mice lacking exons 6 and 7 also display reduced hippocampal NMDA-R function [10]. In addition, pharmacological upregulation of NMDA-R function is suggested to improve ASD symptoms in ASD patients [57, 58] and in mouse models [10, 59–61].

DSCAM is on a short list of 26 genome-wide significant ASD genes in the largest genetic study of ASD to date (Satterstrom et al.; SI Table 2) [62]. All reported ASD cases (*n* = 5), including the patient in our study, attributed to DSCAM are de novo protein truncated variants. In humans, DSCAM is highly expressed during fetal development (9–16 pcw) especially in brain regions, such as the hippocampus, cerebellum, cerebral cortex, and lateral ventricle (https://www.brainspan.org; Brain Span). On cellular level, DSCAM mRNA is highly expressed in neurons, oligodendrocyte progenitor cells and young oligodendrocytes in human and mouse brain (https://www.brainrnaseq.org) [63].

However, how DSCAM mutations actually contribute to the molecular pathogenesis of ASD is largely unknown. Furthermore, little is known about whether DSCAM mutations affect NMDA-R function or not. In this study, for the first time, using forebrain-like neuronal (iN) cells generated from iPSCs of an ASD patient with a de novo mutation in *DSCAM*, we found a reduction in NR1 density and NMDA-R currents, which was restored by overexpressing the full-length DSCAM in ASD iN cells, and recapitulated by overexpressing N-terminal truncated DSCAM or by the knockdown of endogenous DSCAM in control iN cells (Fig. 3, Supplementary Fig. 5), implying a causal link between DSCAM and NMDA-R function.

Our transcriptomic analysis showed that three terms, “trans-synaptic signaling”, “axon development”, and “synapse organization”, out of the top five enriched pathways or terms in the downregulated genes in ASD iN cells were associated with specific neuronal processes. Among these synapse genes, gene expression of NMDA-R subunits was significantly reduced. As a cell adhesion molecule, DSCAM mediates trans-synaptic interactions to promote subsequent organization of post-synaptic receptors and stabilization of presynaptic varicosities during de novo synaptogenesis [25]. Both DSCAM and NMDA-Rs are found in the trans-synaptic membrane, and there is a high probability that they interact with each other. A considerable degree of co-localization between DSCAM and NR1 was revealed in the dendrites of control iN cells, which was significantly reduced in ASD iN cells (Fig. 4). Most of this interaction was observed in dendritic spines, as defined by PSD-95 immunostaining. This DSCAM-NR1-PSD-95 tripartite co-localization further strengthens our hypothesis that reduced NMDA-Rs or DSCAM expression impairs the function of NMDA-R in dendritic spines, which in turn could affect NMDA-R currents in ASD iN cells. Exogenous expression of N-terminal DSCAM resulted in reduced NMDA-R currents in control iN lines, whereas full-length DSCAM rescued NMDA-R currents in ASD iN cells (Fig. 3g). In addition, expression of N-terminal DSCAM caused decreased NR1 expression in control iN cells (Fig. 3h), consistent with our RNA sequencing showing lower level of NMDA-Rs expression in ASD iN cells (Fig. 2). Therefore, the DSCAM mutation (N-terminal truncated DSCAM) might have a dominant-negative property, which plays a role to restrict NMDA-R expression. The DSCAM N-terminal protein may block the NMDA-R active site or its binding sites with DSCAM to restrict NMDA-Rs synaptic expression, which ultimately reduces NMDA-R currents in ASD iN cells. On the other hand, restoring the DSCAM level by overexpressing full-length DSCAM increased NR1 density and NMDA-R currents (Fig. 3g, h). One possible explanation is that overexpression of the full-length DSCAM overcomes the dominant-negative role of the truncated DSCAM, leading to the rescue of NMDA-R currents. Besides, when endogenous DSCAM expression levels were reduced by expressing a specific shRNA, NR1 expression was also decreased in control iN cells (Supplementary Fig. 5b–e). In addition, reintroducing shRNA-resistant DSCAM in control iN cells in which endogenous DSCAM was knocked down by the specific shRNA restored NR1 expression (Supplementary Fig. 5f–i). Therefore, it is also possible that low expression level of NMDA-Rs by reduced DSCAM levels in ASD iN cells impairs NMDA-R currents, as previously discussed. In addition, post-synaptic DSCAM can be activated and generate intracellular signals by interacting with FYN kinase [64], which in turn phosphorylates NMDA-Rs to enhance their function [65, 66]. Therefore, a decreased level of DSCAM or NR1 in ASD iN cells could result in less NMDA-R phosphorylation and reduced currents. Furthermore, DSCAM might play a role as a sensor or linking molecule between Ca^2+^ and effector molecules. Ligand-receptor binding in spines causes ion channel opening for Ca^2+^ entry. Ca^2+^ elevation is spatially restricted to the vicinity of the NMDA-R [67], where sensory molecules, such as the small GTP-binding protein Ras [68] and linking molecules synGAP [69] and RasGRF1 [43] can initiate downstream signaling. We showed that the DSCAM mutation resulted in reduced NMDA-R currents, which could reflect a potential role for DSCAM as a linking molecule to mediate downstream signaling. To validate this, we measured the level of pERK and found a significant reduction in ASD iN cells (Fig. 4). ERK activity in neurons is regulated by diverse extracellular signals via multiple signaling pathways [70]. Agonist binding to the NMDA-R increases post-synaptic Ca^2+^, [71] and activates the ERK pathway. A reduced pERK level in ASD iN cells further suggests a functional role for DSCAM in NMDA-R-mediated intracellular signaling.

Several possibilities exist for how mRNA expression of NMDA-R subunits is decreased in ASD iN cells. First, DSCAM plays a role in organizing NMDA-Rs in dendritic spines by trans-synaptic signaling [25]. DSCAM and other cell adhesion molecules establish the connection between pre- and post-synaptic areas and increase the requirement of NMDA-Rs in spines. Reduction of DSCAM or the presence of truncated N-terminal DSCAM may disturb the trans-synaptic signaling, lowering the demand for NMDA-Rs in spines such that cells produce a smaller number of NMDA-Rs. On the other hand, dendritic levels of DSCAM protein are also regulated by synaptic activity through NMDA-Rs [72]. So, there may be a two-way feedback loop control of NMDA-R expression by DSCAM. Second, the cytoplasmic domain itself or its associated protein of many cell adhesion molecules can translocate to the nucleus to regulate gene expression as a transcriptional regulator [73–75]. Therefore, it could be possible that decreased DSCAM levels may interfere with signaling to the nucleus to regulate gene expression including NMDA-R subunits. In fact, the intracellular domain of DSCAM directly interacts with IPO5, a nuclear import protein which translocates to the nucleus, in turn altering the expression of genes associated with synaptic function and neuronal differentiation [75].

In addition to dysregulated NMDA-R-mediated signaling in ASD iN cells (Fig. 4), we also found downregulation for genes regulating axon development in our RNA sequencing analysis (Fig. 2) with reduced axonal length, but similar dendritic arborization and spine density in ASD iN cells as compared to control (Supplementary Figs. 3 and 7d). Previously, Bruce et al. [76] reported that DSCAM is a potent promoter of axonal outgrowth and in cultures from *Dscam*^del17/del17^ mice, axon outgrowth from the retinal explants was decreased significantly which is consistent with our findings. Others also reported the role of DSCAM in axonal growth, fasciculation, and pathfinding and dendritic field organization in *Drosophila* [23, 77] and mice [78, 79]. We studied human-iN cells with a DSCAM mutation in this regard to provide more valuable insights on the role of DSCAM to regulate neuronal growth and maturation.

We then used the *Dscam*-knockout mouse model to further elucidate the function of DSCAM in ASD pathogenesis. Several *Dscam* KO mice have been reported [37, 80, 81], but social behaviors have not been examined in these mice, partly due to their poor viability against certain genetic backgrounds. We crossed *Dscam* floxed exon 1 mice (B6.129P2-*Dscam*^*tm1.1Kzy*^) [37] with Nestin-Cre mice to generate a conditional *Dscam* KO mouse model. This neural stem cell-specific *Dscam* heterozygous KO mouse showed no gross morphological changes but had deficits in some social interaction behaviors including sociability and social novelty (Fig. 5). However, social communication, as measured by ultrasonic vocalization test, and repetitive behaviors were not altered in Nestin-*Dscam*^+/−^ mice as compared to WT littermates (Supplementary Fig. 9). These results are reminiscent of Chromodomain-helicase-DNA-binding protein 8 (Chd8)-deficient mice, in which social behaviors are defective but repetitive behaviors are normal [82], and they recapitulate the behaviors of our ASD patient, who has a score of 14 in social interaction score and a score of 1 in restricted and repetitive behavior in the ADOS-2. We also detected reduced Dscam expression in mouse neurons (Supplementary Fig. 8) of Nestin-*Dscam*^+/−^ mice consistent with the findings in human heterozygous neurons derived from our ASD patient. These data suggest that the reduced expression of Dscam itself, which is abnormal, is sufficient to elicit an impaired behavioral phenotype in heterozygous Nestin-*Dscam* KO mice. The NMDA/AMPA ratio was reduced in glutamatergic neurons of the ACC in Nestin-*Dscam*^+/−^ mice (Fig. 5), consistent with the decreased NMDA-R currents observed in ASD iN cells (Fig. 3). Taken together, these results suggest that the NMDA-R functional change might be a critical mechanism in DSCAM mutation-related ASD.

In summary, by using human ASD patient-derived iN cells and a *Dscam* mutant mouse model, we revealed a novel ASD pathophysiology in that the DSCAM mutation may lead to autistic phenotypes by impairing NMDA-R function.

## Supplementary information

Supplemental Information
