# Supplementary material for: Dysfunction of NMDA receptors in neuronal models of an autism spectrum disorder patient with a DSCAM mutation and in Dscam-knockout mice
Source: Mol Psychiatry. 2021 Jul 12;26(12):7538–49. doi: 10.1038/s41380-021-01216-9 (PMC8873012; doi:10.1038/s41380-021-01216-9)
Supplement: Supplementary file 1 — Supplemental Information [file 41380_2021_1216_MOESM1_ESM.docx]

***Supplemental Information***

***Article***

**Dysfunction of NMDA receptors in neuronal models of an Autism Spectrum Disorder patient with a *DSCAM* mutation** **and in *Dscam* knockout mice**

Chae-Seok Lim^1,2,*^, Min Jung Kim^1,*^, Ja Eun Choi^1,*^, Md Ariful Islam^1,*^, You-Kyung Lee^3*^, Yinyi Xiong^2^, Kyu Won Shim^4^, Jung-eun Yang^1^, Ro Un Lee^1^, Jiah Lee^1^, Pojeong Park^1^, Ji-Hye Kwak^5^, Hyunhyo Seo^5^, Chul Hoon Kim^7^, Jae-Hyung Lee^7^, Yong-Seok Lee^8^, Su-Kyeong Hwang^9^, Kyungmin Lee^5,**^, Jin-A Lee^3,**^, and Bong-Kiun Kaang^1, **^

^1^School of Biological Sciences, Seoul National University, Seoul 08826, South Korea, ^2^Department of Pharmacology, Wonkwang University School of Medicine, Jeonbuk 54538, South Korea, ^3^Department of Biotechnology and Biological Sciences, Hannam University, Daejeon 34430, South Korea, ^4^Interdisciplinary Program in Bioinformatics, Seoul National University, Seoul 08826, South Korea, ^5^Department of Anatomy, School of Medicine, Kyungpook National University, Daegu 41944, South Korea, ^6^Department of Pharmacology, Yonsei University College of Medicine, Seoul 03722, South Korea, ^7^Department of Life and Nanopharmaceutical Sciences, Department of Oral Microbiology, Kyung Hee University School of Dentistry, Seoul 02447, South Korea, ^8^Department of Physiology, Biomedical Sciences, Neuroscience Research Institute, Seoul National University College of Medicine, Seoul 03080, South Korea, ^9^Department of Pediatrics, School of Medicine, Kyungpook National University, Daegu 41944, South Korea.

^*^These authors contributed equally to this work.

**^**^Corresponding authors:**

*Kyungmin Lee, M.D., Ph.D.*

Department of Anatomy, School of Medicine, Kyungpook National University, 2-101, Dongin-dong, Jung-gu, Daegu 41944, South Korea

E-mail: irislkm@knu.ac.kr; Phone: +82-53-420-4803; Fax: +82-53-427-1468

*Jin-A Lee, Ph.D.*

Department of Biotechnology and Biological Sciences, Hannam University, Bldg 720 Rm 1206, 1646 Yuseong-daro, Yuseong-gu, Daejeon 34430, South Korea

E-mail: leeja@hnu.kr; Phone: +82-42-629-8785; Fax: +82-42-629-8769

*Bong-Kiun Kaang, Ph.D.*

School of Biological Sciences, Seoul National University, Bldg 504 Rm 202, 1 Gwanangno, Gwanak-gu, Seoul 08826, South Korea

E-mail: kaang@snu.ac.kr; Phone: +82-2-880-7525; Fax: +82-2-884-9577

**Running title:** NMDA-R dysfunction in a *DSCAM*-mutated ASD patient

**Supplementary Materials and Methods**

**Whole exome sequencing and de novo variant calling**

Whole blood was obtained from the patient and his family members (father, mother, and brother) after informed consent for the protocol (KNUH 2013-07-011-004) guided by the KNUH IRB. Whole exome sequencing was performed as described by Hwang et al [^1^](#_ENREF_1). For each sample, approximately 27–31 million read pairs were generated. The generated raw sequencing reads were assessed and trimmed using the Sickle program (v. 1.33) to ensure the quality of the raw reads. Pre-processed reads were mapped to the reference human genome sequence GRCh37 using the Burrow-Wheeler Aligner (BWA, version 0.7.10) [^2^](#_ENREF_2). To reduce potential bias caused by the sequencing processes, the mapped duplicated reads were marked using Picard (v. 1.118). Insertion and deletion (INDEL) realignment and base quality recalibration were performed using GATK (version 3.2.2) [^3^](#_ENREF_3). Using the alignments, small nucleotide variants (SNVs) and small INDELs were called by the GATK HaplotypeCaller, and called variants were filtered using the GATK variant quality score recalibration process. *De novo* variants were identified by Triodenovo (v. 0.04) [^4^](#_ENREF_4) and further filtered to exclude those included in dbSNP142, 1000 Genomes Project (Oct 2014), NHLBI-ESP project with 6500 exomes, or ExAC 65,000 exomes at the level of 1% minor allele frequency (MAF) by ANNOVAR [^5^](#_ENREF_5). In addition, the *de novo* variants were filtered out if they were present in the in-house genome and exome databases.

**Generation and characterization of iPSCs**

All experimental procedures for fibroblasts and iPSCs were approved by the Kyungpook National University Hospital IRB (KNUH 2013-07-011-004) and the Hannam University IRB (HANNAM 2013-12k). Patient-specific ASD iPSC lines (ASD#3, ASD#4, or ASD#5) were generated from skin fibroblasts of the 5-year-old boy (now 12-year-old) with ASD symptoms, and a sibling control iPSC line (Control#3) was generated from skin fibroblasts of his 3-year-old male sibling (now 10-year-old) without ASD symptoms (Fig. 1a, Supplementary Fig. 1) using the integration-free method as previously described [^6^](#_ENREF_6). Other control iPSC lines (Control#1 and Control#2) were also generated from skin fibroblasts of a 37-year-old healthy female or 8-year-old healthy boy, respectively. Cultured fibroblasts from skin biopsies were transfected with episomal vectors containing OCT3/4, shp53 RNA, SOX2, L-MYC, or LIN28 via electroporation (Invitrogen, Neon^TM^ transfection system, MP922114). Seven days after transfection, fibroblasts were trypsinized and re-seeded onto a feeder layer of mouse embryonic fibroblasts (MEFs). The cells were maintained in an embryonic stem cell (ESC) medium containing 20% knockout serum, β-mercaptoethanol (Gibco, 21985023), 1x GlutaMAX (Gibco, 35050-061), 1x MEM-NEAA (Gibco, 11140050), penicillin/streptomycin (Hyclone, SV30010), and DMEM-F12 (Gibco, 11320-033). Three to four weeks after iPSC induction, the iPSC-like colonies were picked and transferred onto new feeder layers. After passage 5, iPSC colonies were transferred into a feeder-free cell culture dish coated with vitronectin (Gibco, A14700) and maintained in Essential 8 media (Gibco, A1517001) supplemented with Essential 8 supplement and penicillin/streptomycin.

To characterize each iPSC line, the expression levels of pluripotent markers were analyzed by RT-PCR (Sox2, Nanog, Rex1, or Oct3/4) and by immunocytochemistry using antibodies (Oct3/4, Nanog, SSEA4, TRA-1-60, or TRA-1-81). Alkaline phosphatase staining was performed to evaluate the stemness of each iPSC clone according to the manufacturer’s instructions (Alkaline phosphatase staining kit [AP100R-1], System Biosciences). Karyotype analysis using each iPSC line was performed by GTG-banding analysis to confirm a normal karyotype (Gendix Inc., Korea)

**Neuronal induction from iPSCs**Induced neuronal (iN) cells were generated as described previously [^7^](#_ENREF_7), with minor modifications. First, culture plates were coated with Matrigel (Corning, 354230), iPSCs were dissociated with Accutase (Innovative Cell Technologies, AT104), and cells were plated at a density 80,000 cells/cm^2^ in mTeSR1 medium (w/ supplement) (Stemcell Technologies, 85850) containing 10 μM Y-27632 (Tocris, 1254). The culture media was replaced the next day with fresh mTeSR1 (w/ supplement) containing rtTA+Ngn2 lentiviruses. On day 0, 2 mg/mL doxycycline (Sigma, D9891) was added to induce TetO gene expression after replacing the culture medium with DMEM/F12 medium (Gibco, 11320033) supplemented with 1% N2 (Gibco, 17502-048), 1% MEM nonessential amino acids (Gibco, 11140050), 10 ng/mL human BDNF (PeproTech; 450-02), 10 ng/mL human NT-3 (PeproTech; 450-02), and 0.2 μg/ml mouse laminin (Sigma, L2020). On day 1, 1 μg/mL puromycin (Merck, 540411) was added to the culture for 24 hours to select virus-infected cells. On day 2, the culture medium was replaced again with a 1:1 mixture of glia-conditioned medium (GCM) and Neurobasal medium (Gibco, 21103-049) supplemented with B27 (Gibco, 17504-044) and Glutamax (Gibco, 35050-061). Additionally, 10 ng/mL BDNF, 10 ng/mL NT3, 2 mg/mL doxycycline, and 1 μg/mL puromycin were added to the culture. GCM was prepared by incubating mouse glial cells with Neurobasal medium supplemented with B27 and Glutamax for 24 hours. From day 4~8, 50% of the medium was replaced every other day. One to two μmol of cytosine-β-*d*-arabino-furanoside (Sigma, C-6645) was added to the cultures when other proliferating cell types were present. On day 9, neurons were dissociated with 0.25% Trypsin (Hyclone, SH30042.01) and plated at 30,000 cells/cm^2^ on Matrigel-coated coverslips containing a monolayer of mouse glial cells in Neurobasal medium supplemented with B27, Glutamax, BDNF, NT3, doxycycline, and 2.5% fetal bovine serum (FBS, WelGENE, S001‑01). Neurons were transfected with the indicated plasmids using Lipofectamine^®^ 2000 reagent (Invitrogen, 11668-019) using the manufacturer’s protocol.

**Lentivirus generation**

Lentiviruses for converting iPSC lines to induced neuronal (iN) cells were prepared according to a previous paper [^7^](#_ENREF_7), with some modifications. In brief, Lenti-X 293T cells (Clontech, 632180) were co-transfected with Ngn2-expressing plasmid (13.3 μg), packaging plasmid (psPAX2, 10 μg), and VSV-G envelope plasmid (pMD2.G, 3.3 μg) using the calcium phosphate method. Six to eight hours after transfection, cultures were washed with DMEM and given fresh DMEM with 10% FBS, 2 mM L-Glutamine, and penicillin/streptomycin. Supernatant containing viruses was harvested 72 hours after transfection, centrifuged (20,000 rpm for 2 h), and filtered with a 0.22 μm syringe filter (Sartorius, 16534). Viral pellets were resuspended with 200 μL of DMEM/F12 (Gibco, 11320-033). The viral titer was determined by measuring the GFP expression level after treatment with the viruses to Lenti-X 293T cells.

***hDSCAM* plasmid construction**

P2A-mRuby2 was PCR-amplified from the pztv-DDvenus-P2A-mRuby2 plasmid (Forward primer: 5’-AGT TAT GGC GCG CCG CTA ACT TCA GCC TGC T-3’; Reverse primer: 5’-AGT TAT GCT AGC CTT GTA CAG CTC GTC CAT CC-3’) and inserted into the pLenti vector at AscI and NheI sites. The LoxP sequence at the 3’ end of the P2A-mRuby2 sequence was removed by Klenow reaction after digesting the plasmid with EcoRI and NheI. Then, full-length and N-terminal truncated (to mimic the L684X mutation) DSCAM sequences were PCR-amplified from pcDNA3-hDSCAM-FLAG (a gift from Dr. Guofa Liu, The University of Toledo) as a template (for full length DSCAM: Forward primer, 5’- GAG GTA CCG GAT CCT CTA GAA TGT GGA TAC TGG CTC TCT C-3’; Reverse primer, 5’- TGA AGT TAG TAG CGG CGC GCC TAC CAG GGT GTA AGA TTT TG-3’; for N-terminal [L684X] DSCAM: Forward primer, 5’- GAG GTA CCG GAT CCT CTA GAA TGT GGA TAC TGG CTC TCT C-3’; Reverse primer, 5’- TGA AGT TAG CGG CGC GCC CTG GCT TTG GTG CTC CAC AG-3’). PCR products were inserted into pLenti-P2A-mRuby2 using the EZ-Fusion™ Cloning Kit (Enzynomics, EZ015).

For the control experiments, the pLenti-mRuby2 plasmid was prepared from pLenti-P2A-mRuby2 plasmid (from the first step) by removing the P2A sequence. EcoRI and XbaI enzymes were used for vector plasmid digestion. The PCR product for the mRuby2 sequence was generated using following primers and pLenti-P2A-mRuby2 plasmid (from the first step) as template. The PCR product was inserted into the digested vector using EZ-Fusion™ Cloning Kit (Enzynomics, EZ015): Forward primer, 5’- TGA GGT ACC GGA TCC TCT AGA TGG TGT CTA AGG GCG AAG AGC TGA-3’; Reverse primer, 5’- GAT AAG CTT GAT ATC GAA TTT CAC TTG TAC AGC TCG TCC ATC C-3’.

***hDSCAM*-specific shRNA and shRNA-resistant *hDSCAM* plasmids construction**

Regarding DSCAM knock-down experiments, shRNA (hDSCAM-4503-shRNA: 5’-GCAGAAGACTCCGGCTATTAC-3’) was designed using Invitrogen shRNA designing tools (http://rnaidesigner.thermofisher.com/rnaiexpress/design.do) and inserted into pSuper-neo-dTomato vector (Addgene). For control shRNA, three nucleotides from the shRNA sequences were mutated. To generate a shRNA-resistant full-length DSCAM construct, three nucleotides from the shRNA target sequences were mutated (5'-GCAGAgGAtTCgGGCTATTAC-3', lowercase: mutated nucleotides) using a mutagenesis kit (QuikChangeII XL Site-Directed Mutagenesis kit, Agilent, #200522), resulting in pcDNA3-hDSCAM(R)-FLAG. Mutations were then confirmed by sequencing.

**Quantitative RT-PCR**

To analyze the gene expression of each iPSC clone, total mRNA was extracted using Trizol (MRC, TR118) or the Monarch Total RNA Miniprep Kit (T2010S), and cDNA was synthesized using a Superscript III reverse transcription kit (Invitrogen, 18018-093; according to the manufacturer’s instructions) or LunaScript RT Super Mix kit (E3010L). PCR was performed using specific primers presented in the Supplementary Tables, including Sox2 (Forward: 5’-GGG AAA TGG GAG GGG TGC AAA AGA GG-3’; Reverse: 5’-TTG CGT GAG TGT GGA TGG GAT TGG TG-3’), Nanog (Forward: 5’-AAG ACA AGG TCC CGG TCA AG-3’; Reverse: 5’-CAGGCATCCCTGGTGGTAG), Rex1 (Forward: 5’-AAG GCA AGT CAA GCC AAG ACC-3’; Reverse: 5’-TTC CAA AGA ACA TTC AAG GGA GC-3’), and Oct3/4 (Forward: 5’-CCC CAG GGC CCC ATT TTG GTA CC-3’; Reverse: 5’-ACC TCA GTT TGA ATG CAT GGG AGA GC-3’). Real-time PCR was conducted using Taqman probes (Sox2 (Thermo, Hs00602736_s1), Nanog (Thermo, Hs02387400_g1), or Oct3/4 (Thermo, Hs00742896_s1)) with a LunaScript RT Super Mix kit (Applied Biosystems).

To analyze gene expression changes in the iN cells, quantitative RT-PCR was conducted with mRNAs extracted from 2-week-old iN cell cultures. RNA purification was performed with a Takara MiniBEST universal RNA extraction kit (Takara, #9767A) and cDNA was synthesized using a PrimeScript™ 1st strand cDNA Synthesis kit (Takara, #6110A). The PCR was performed using the specific primers presented in the Supplementary Tables.

**Transfection**

To evaluate the knock-down efficiency of the *hDSCAM* shRNAs, pSuper-H1-hDSCAM-shRNA-dTomato and pcDNA3-hDSCAM-FLAG constructs were co-transfected in LX293T cells using Lipofectamine 2000 or 3000 (ThermoFisher, #11668027 or #L3000008) and western blot analysis was performed using the cell lysates of the LX293T cells 2 days after the transfection.

**Western blot analysis**

Western blot analysis was performed as previously described. Briefly, brain samples were homogenized in RIPA buffer containing protease inhibitor cocktail (Roche, 11836153001). With respect to iN cells, 6-week cultures were washed with PBS and then homogenized in RIPA buffer. Samples were prepared after measuring protein levels using BCA quantification. Equal amounts of protein samples were separated by SDS-PAGE gel electrophoresis and transferred on a membrane. The membrane was then blocked with 5% skim milk and incubated with the following primary antibodies: DSCAM (LsBio, LS-B5787, 1:1,000), NR1 (abcam, 109182, 1:500), and Tuj1 (Convance, MMS-435, 1:1,000) followed by incubation of the appropriate secondary antibodies. Chemiluminescent signals were detected and quantified using a ChemiDoc^TM^ MP device (Bio-Rad).

**Immunocytochemistry**

To examine the expression of stem cell markers in each iPSC clone, fixed cells were permeabilized with 0.1% Triton X-100 and blocked with 3% BSA. Primary antibodies (Oct3/4 (Santa Cruz, sc-5279), Nanog (Reprocell, RCAB003P-F), SSEA4 (abcam, ab16287), TRA-1-60 (Millipore, MAB4360), and TRA-1-81 (Millipore, MAB4381) were incubated at 4°C overnight, followed by incubation with secondary antibodies (Alexa Fluor 488 or Cy3-conjugated antibody) at RT for 1.5 h. Cell images were taken by a confocal microscope (Zeiss, LSM-880). For iN cells, 2-week- or 6-week-old iN cells were fixed by serial incubation with 4% paraformaldehyde and methanol [^8^](#_ENREF_8). Fixed cells were blocked (5% normal goat serum, 0.05% Triton X-100 in PBS, pH 7.4), and then treated with primary antibodies MAP2 (Millipore, ab5622), phosphor-ERK1/2 (Cell Signaling, 9109), NR1 (BD Biosciences, 556308 or Synaptic Systems, 114011), DSCAM (LSBio, B5787), and tubulin (Sigma, T4026) followed by Alexa Fluor-conjugated secondary antibodies (Invitrogen). Three-dimensional (3D) reconstruction of neurites and quantification of immunostained puncta were performed using IMARIS (Bitplane, Zurich, Switzerland) software. Experimenters were blinded to the immunocytochemical samples until completing quantitative analyses.

**Proximity ligation assay (PLA)**

Generic *in situ* PLA was performed using a Duolink kit (Sigma Aldrich, DUO92101) according to the manufacturer’s instructions with minor changes. Induced neuronal (iN) cells were fixed with sequential paraformaldehyde/methanol treatment and blocked with buffer (5% normal goat serum, 0.05% Triton X-100 in PBS, pH 7.4). Next, cells were incubated with primary antibodies overnight at 4°C, followed by secondary antibodies conjugated with oligonucleotides (PLA probe anti-mouse MINUS and PLA probe anti-rabbit PLUS) for 2 h at 37°C in a humidified chamber, and cells were then incubated with ligase for 30 min at 37°C. After hybridization and ligation of the DNA oligonucleotides, amplification solution along with polymerase was added. The amplified product was detected as a red signal using complementary fluorescently labeled oligonucleotides. The PLA reactions were followed by immunocytochemistry, the addition of primary antibodies (PSD-95, NeuroMab, 75-028), and fluorophore-conjugated secondary antibodies.

**Electrophysiology**

Whole cell patch clamp recordings from iN cells were performed as previously described [^7^](#_ENREF_7) at room temperature while perfusing with a bath solution containing 140 mM NaCl, 5 mM KCl, 10 mM HEPES, 2 mM CaCl_2_, 2 mM MgCl_2_, and 10 mM glucose at pH 7.4. Membrane excitability was recorded at a membrane potential of -70 mV in current clamp mode, during which stepwise currents were injected for 500 ms to elicit action potentials; current steps ranging from 0–60 pA were delivered at 10-pA increments. The pipette solution used for these recordings contained 145 mM K-gluconate, 5 mM NaCl, 10 mM HEPES, 1 mM MgCl_2_, 0.2 mM EGTA, 2 mM MgATP, and 0.1 mM Na_3_GTP (280 ~ 300 mOsm, adjusted to pH 7.2 with KOH). Miniature excitatory postsynaptic currents (mEPSCs) were recorded in voltage-clamp mode in the presence of picrotoxin (100 μM) and tetrodotoxin (1 μM). The internal solution used for recording contained 100 mM Cs-gluconate, 5 mM NaCl, 10 mM HEPES, 10 mM EGTA, 20 mM TEA-Cl, 3 mM QX-314, 4 mM MgATP, and 0.3 mM Na_3_GTP (280~300 mOsm, pH adjusted to 7.2 with CsOH). Only cells with a <25% change in access resistance were included in the analysis with MiniAnalysis program (Synaptosoft). NMDA currents (*I_NMDA_*) isolated with picrotoxin (100 μM) and CNQX (20 μM) were recorded in voltage-clamp mode in a bath containing 140 mM NaCl, 5 mM KCl, 10 mM HEPES, 2 mM CaCl_2_, 0.8 mM MgCl_2_, and 10 mM glucose (pH 7.4). NMDA-R-mediated currents were evoked by puff application (5 psi) of 100 μM NMDA and 10 μM glycine using 3–5 MΩ glass pipettes.

To measure the NMDA/AMPA ratio, coronal brain slices (300 μm) were prepared from adult mice using a vibratome (Leica, VT1000S) in ice-chilled slicing solution that contained (mM): 93 NMDG, 2.5 KCl, 30 NaHCO_3_, 1.2 NaH_2_PO_4_, 20 HEPES, 10 MgSO_4_, 25 D-glucose, 5 sodium ascorbate, 2 Thiourea, 3 sodium pyruvate, and 0.5 CaCl_2_, saturated with 95% O_2_ and 5% CO_2_. The slices were transferred to an incubation chamber containing the recording solution (artificial cerebrospinal fluid, ACSF; mM): 124 NaCl, 3 KCl, 26 NaHCO_3_, 1.25 NaH_2_PO_4_, 2 MgSO_4_, 10 D-glucose and 2 CaCl_2_ (carbonated with 95% O_2_ and 5% CO_2_). Slices recovered at 32–34°C for 10 min, and then maintained at 26–28°C for a minimum of 1 h before recordings were made. Whole-cell recording was performed at 32°C during continuous perfusion at 3–4 mL/min with ACSF containing 50 μM picrotoxin (HelloBio) to prevent GABA_A_R transmission. Pyramidal cells in the ACC layer 2/3 were visualized with IR-DIC optics (Olympus), and afferent pathways from layer 1 were stimulated at a frequency of 0.1 Hz. The whole-cell solution was comprised of (mM): 5 NaCl, 100 Cs-gluconate, 10 HEPES, 10 EGTA, 4 Mg-ATP, 0.3 Na_3_-GTP, 3 QX-314 and 20 TEA-Cl. The pH was adjusted to 7.2–7.3 with CsOH and osmolarity was set to 285–290 mOsm/L. Borosilicate glass pipettes were used with a resistance of 3–5 MΩ, and experiments were only accepted for analysis if the series resistance values were <20 MΩ and varied by 10% during the course of experiment. Signals were filtered at 10 kHz and digitized at 20 kHz using Multiclamp 700B (Molecular Devices). The peak amplitude of evoked EPSCs (pA) was monitored and analyzed using WinLTP and Clampfit. Cells were clamped at a holding potential (V_h_) of -70 mV to measure the peak of AMPAR-mediated synaptic transmission. NMDA-R currents were estimated at 60 ms after the stimulation onset (V_h_ = +40 mV). Averages of 10 consecutive responses obtained at these holding potentials were used for the calculation of NMDA/AMPA ratio. Experimenters were always blinded to the genotypes or cell identities.

**Behavioral analyses**

Behavioral analyses were performed as previously described [^9-12^](#_ENREF_9). All mouse behavioral experiments were performed using male mice 14–26 weeks of age. Genotypes of mice were randomized by other experimenters who did not perform the experiments, so that the experimenters were always blinded to the genotypes. Behavioral tasks were conducted in the following order: open field test, marble burying test, repetitive behavior test, reciprocal interaction test, three-chamber test, and ultrasonic vocalization *(*USV) test. According to our pre-established criteria, mice with any injury before or during experiments were excluded from the behavioral analyses.

*Open-field test:* Mice were placed in the center of the open-field apparatus (40 cm x 40 cm x 40 cm square-shaped box) and allowed to freely move in the open field for 30 min under dim light. Mouse movements were analyzed by Ethovision 9.0 (Noldus).

*Marble burying test:* Mice were allowed to freely move for 30 min in a cage with 20 marbles that were located at even intervals on a beta chip (Orient). The number of buried marbles was manually counted every 5 min.

*Repetitive behavior test:* Mice were placed in a new cage with new bedding, and their behaviors were recorded for 20 min. Time spent on repetitive behaviors (self-grooming, rearing, and sniffing) was manually quantified during the last 10 min.

*Reciprocal interaction test:* A pair of test mice (WT and WT, WT and KO, and KO and KO) were placed in a new cage with new bedding. The reciprocal interaction behaviors of each pair were recorded for 10 min. Time spent on interactions (sniffing, following, nose poking) was manually determined.

*Three-chamber test:* Test mice and stranger mice were first habituated separately for two consecutive days in an empty apparatus containing three identical chambers in a row. For the first 10 min, mice were allowed to move freely in the apparatus without any object or stranger mouse. Then, the test mouse was allowed to move around the entire apparatus for 10 min with an object located on one side of the chamber and a stranger mouse placed on the other side of the chamber (for the sociability test). Finally, the test mouse was allowed to move around the apparatus for 10 min with a familiar mouse located on one side of the chamber and an unfamiliar mouse placed on the other side of the chamber (for the social recognition test). Time spent exploring the object or the stranger mouse was analyzed manually.

*Ultrasonic vocalization test:* Test mice were single-caged for 7 days. After habituating the test mice for 5 min in a new cage with new bedding, a female in estrous was put into the cage. Ultrasonic vocalization between the test mouse and the encountered female mouse was recorded, and the total number of calls and latency to the first call were analyzed by Avisoft software.

**Data availability**

The RNA sequencing data have been deposited at NCBI GenBank under BioProject ID PRJNA670927 (BioSample SAMN16523537 - SAMN16523540). Please check data using the private reviewer link, https://dataview.ncbi.nlm.nih.gov/object/PRJNA670927?reviewer=p475a6p34a3ftl7ep3r68i5s2v.

**References**

1. Hwang SK, Lee JH, Yang JE, Lim CS, Lee JA, Lee YS *et al.* Everolimus improves neuropsychiatric symptoms in a patient with tuberous sclerosis carrying a novel TSC2 mutation. *Mol Brain* 2016; **9**(1)**:** 56.

2. Li H, Durbin R. Fast and accurate short read alignment with Burrows-Wheeler transform. *Bioinformatics* 2009; **25**(14)**:** 1754-1760.

3. McKenna A, Hanna M, Banks E, Sivachenko A, Cibulskis K, Kernytsky A *et al.* The Genome Analysis Toolkit: a MapReduce framework for analyzing next-generation DNA sequencing data. *Genome Res* 2010; **20**(9)**:** 1297-1303.

4. Wei Q, Zhan X, Zhong X, Liu Y, Han Y, Chen W *et al.* A Bayesian framework for de novo mutation calling in parents-offspring trios. *Bioinformatics* 2015; **31**(9)**:** 1375-1381.

5. Wang K, Li M, Hakonarson H. ANNOVAR: functional annotation of genetic variants from high-throughput sequencing data. *Nucleic Acids Res* 2010; **38**(16)**:** e164.

6. Okita K, Matsumura Y, Sato Y, Okada A, Morizane A, Okamoto S *et al.* A more efficient method to generate integration-free human iPS cells. *Nat Methods* 2011; **8**(5)**:** 409-412.

7. Zhang Y, Pak C, Han Y, Ahlenius H, Zhang Z, Chanda S *et al.* Rapid single-step induction of functional neurons from human pluripotent stem cells. *Neuron* 2013; **78**(5)**:** 785-798.

8. Moon IS, Cho SJ, Jin I, Walikonis R. A simple method for combined fluorescence in situ hybridization and immunocytochemistry. *Mol Cells* 2007; **24**(1)**:** 76-82.

9. Won H, Lee HR, Gee HY, Mah W, Kim JI, Lee J *et al.* Autistic-like social behaviour in Shank2-mutant mice improved by restoring NMDA receptor function. *Nature* 2012; **486**(7402)**:** 261-265.

10. Kim MJ, Lee RU, Oh J, Choi JE, Kim H, Lee K *et al.* Spatial Learning and Motor Deficits in Vacuolar Protein Sorting-associated Protein 13b (Vps13b) Mutant Mouse. *Exp Neurobiol* 2019; **28**(4)**:** 485-494.

11. Lim CS, Kim H, Yu NK, Kang SJ, Kim T, Ko HG *et al.* Enhancing inhibitory synaptic function reverses spatial memory deficits in Shank2 mutant mice. *Neuropharmacology* 2017; **112**(Pt A)**:** 104-112.

12. Lim CS, Nam HJ, Lee J, Kim D, Choi JE, Kang SJ *et al.* PKCalpha-mediated phosphorylation of LSD1 is required for presynaptic plasticity and hippocampal learning and memory. *Sci Rep* 2017; **7**(1)**:** 4912.

**Supplementary Figures and Figure Legends**

**
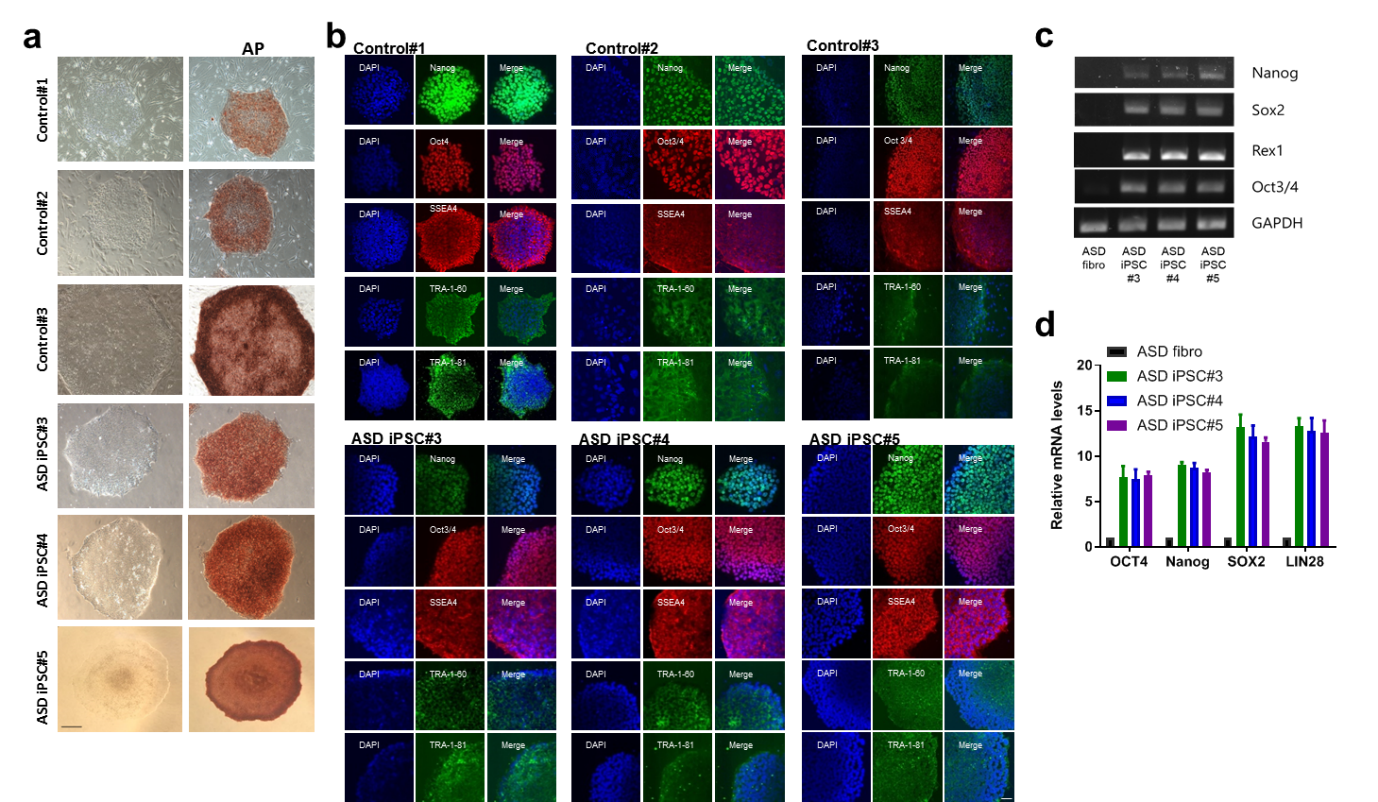
**

**Supplementary Figure 1. Characterization of human induced pluripotent stem cells (iPSCs) derived from fibroblasts.**

(a) Morphology of iPSC colonies and alkaline phosphatase staining in iPSCs. Scale bar: 200 μm. (b) Immunostaining of human iPSCs with specific antibodies against intracellular pluripotent stem cell markers (Oct3/4, SSEA3, SSEA4, Tra1-60, and Tra1-81). Scale bar: 50 μm. (c-d) RT-PCR analysis of pluripotency marker expression (Oct3/4, Sox2, Rex1, and Nanog) in iPSCs. Data in the bar graph are presented as the mean ± SEM.

**
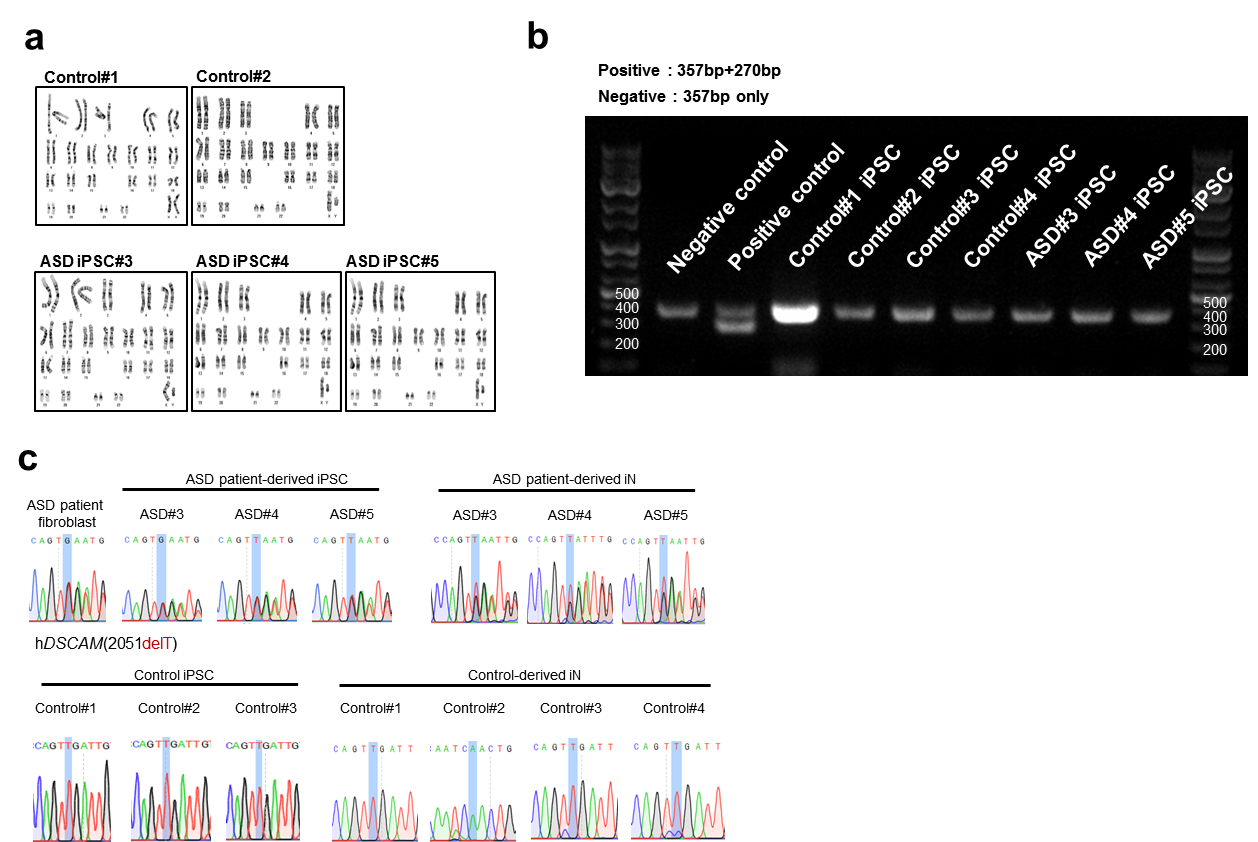
 Supplementary Figure 2. Chromosome stability and genomic sequence of the *DSCAM* gene deletion mutation (2051del(T)) in fibroblasts and iPSC-derived neurons.**

(a) All iPSC lines used had normal karyotypes. (b) All control iPSC (#1, 2, 3, and 4) and ASD iPSC (#3, 4, and 5) lines were negative for mycoplasma. (c) Genomic sequence of the *DSCAM* gene in ASD fibroblasts (or control fibroblasts) and iPSC-derived neurons showing the 2051del(T).

**
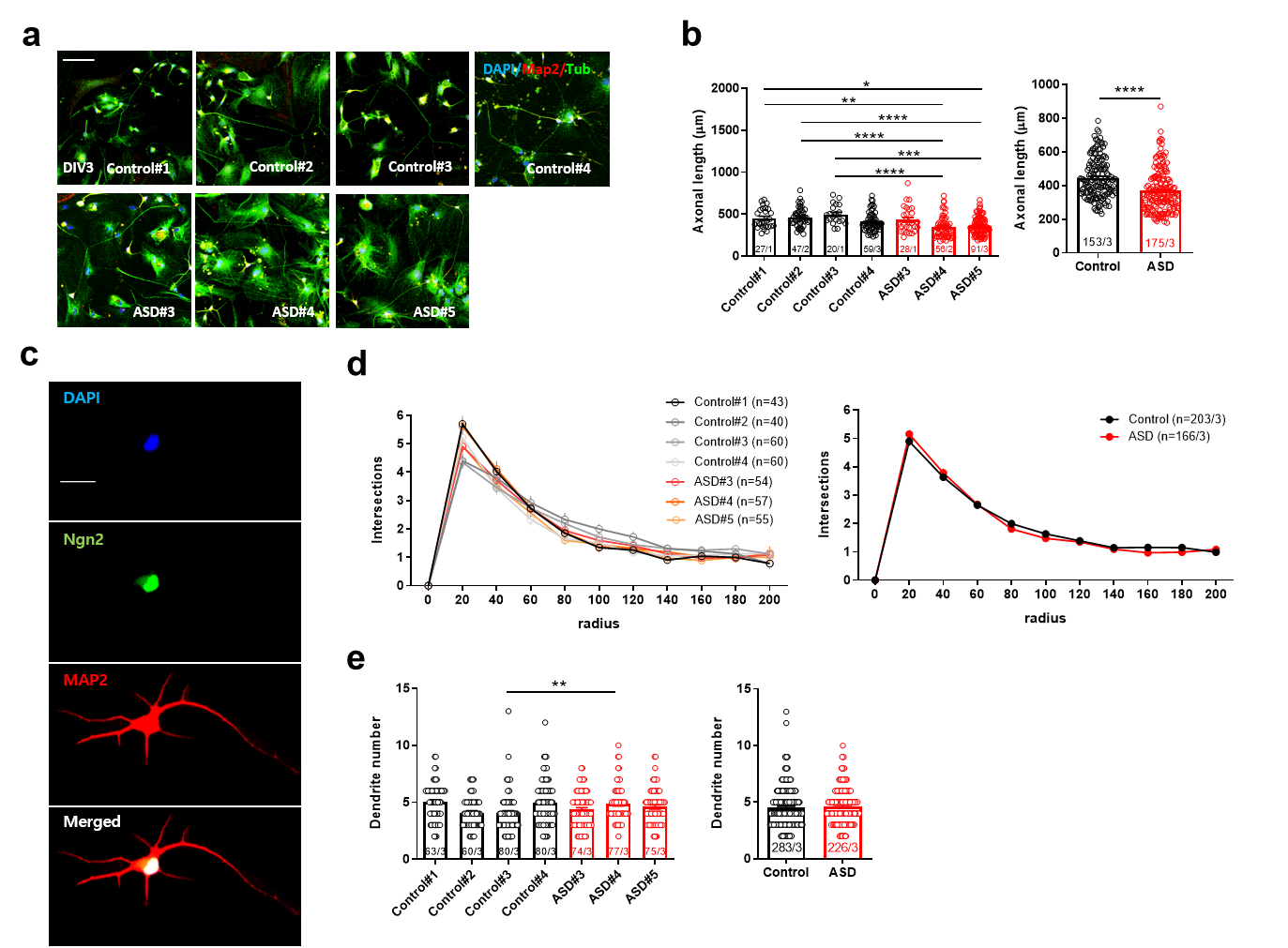
**

**Supplementary Figure 3. Neuronal growth analyses of iN cells.**

(a) Representative images of axon at day3. Tubulin staining shows long axons without Map2 signal. Scale bar: 100 μm. (b) Axonal length was decreased in ASD iN cells [Control: n = 153, ASD: n = 175 (Control#1: n = 27, Control#2: n = 47, Control#3: n = 20, Control#4: n = 59, ASD#3: n = 28, ASD#4: n = 56, ASD#5: n = 91), Kruskal-Wallis test for left panel (*p* < 0.0001) followed by Dunn's multiple comparisons test (Control#1 vs ASD#4, ***p* < 0.01, Control#1 vs ASD#5, **p* < 0.05, Control#2 vs ASD#4, *****p* < 0.0001, Control#2 vs ASD#5, *****p* < 0.0001, Control#3 vs ASD#4, *****p* < 0.0001, Control#3 vs ASD#5, ****p* < 0.001), Mann-Whitney test for right panel (*****p* < 0.0001)]. (c) Representative images of MAP2 staining. Scale bar: 30 μm. (d) Dendritic Sholl analysis showed no difference between control and ASD iN cells [Control: n = 326, ASD: n = 226 (Control#1: n = 63, Control#2: n = 60, Control#3: n = 80, Control#4: n = 80, ASD#3: n =74, ASD#4: n = 77, ASD#5: n = 75); two-way repeated-measures ANOVA, interaction, F (10, 4781) = 1.756, *p* = 0.0632]. (e) The number of dendrites were comparable between control and ASD iN cells [Control: n = 283, ASD: n = 226 (Control#1: n = 63, Control#2: n = 60, Control#3: n = 80, Control#4: n = 80, ASD#3: n = 74, ASD#4: n = 77, ASD#5: n = 75), Kruskal-Wallis test for left panel (*p* < 0.0001, Control#1 vs Control#2, **p* < 0.05, Control#1 vs Control#3, ****p* < 0.001, Control#3 vs Control#4, ***p* < 0.01, Control#3 vs ASD#4, ***p* < 0.01), Mann-Whitney test for right panel, *p* = 0.3512)].


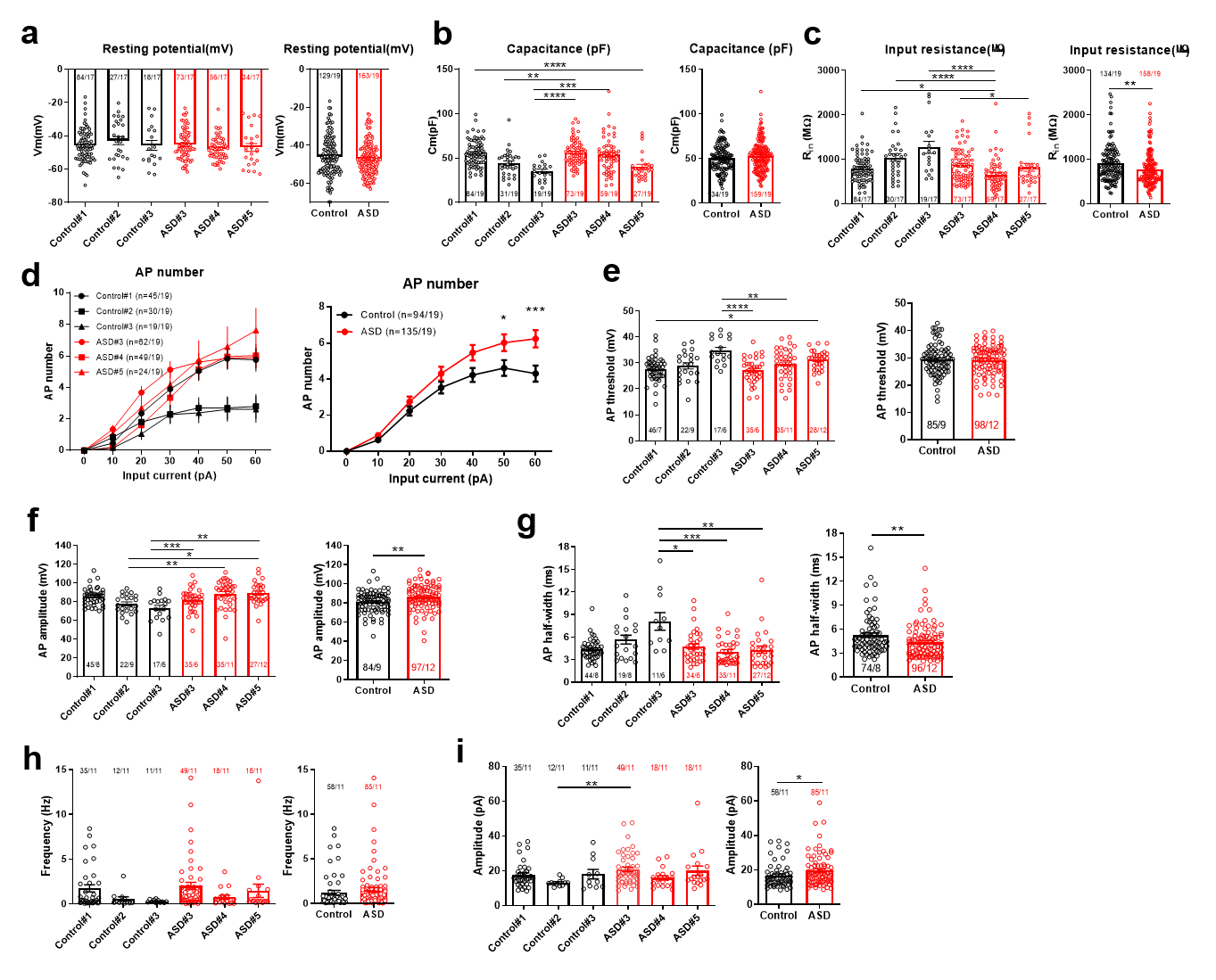


**Supplementary Figure 4. Basic electrophysiological properties and synaptic transmissions of iN cell lines.**

(a) Resting membrane potential is unchanged in ASD iN cells as compared to control iN cells [Control: n = 129, ASD: n = 163 (Control#1: n= 84, Control#2: n= 27, Control#3: n= 18, ASD#3: n= 73, ASD#4: n= 56, ASD#5: n= 34), one-way ANOVA for left panel (interaction, *p* = 0.4104); Mann-Whitney test, *p* = 0.4613]. (b) No difference in capacitance [Control: n = 134, ASD: n = 159 (Control#1: n= 84, Control#2: n= 31, Control#3: n= 19, ASD#3: n= 73, ASD#4: n= 59, ASD#5: n= 27), Kruskal-Wallis test for left panel (*p* < 0.0001) followed by Dunn's multiple comparisons test (Control#1 vs ASD#5, *****p* < 0.0001, Control#2 vs ASD#3, ***p* < 0.01, Control#3 vs ASD#3, *****p* < 0.0001, Control#3 vs ASD#4, ****p* < 0.001); unpaired *t*-test, *p* = 0.1487]. (c) Input resistance was similar between control iN cells and ASD iN cells [Control: n = 134, ASD: n = 158 (Control#1: n= 84, Control#2: n= 31, Control#3: n= 19, ASD#3: n= 72, ASD#4: n= 59, ASD#5: n= 27), Kruskal-Wallis test for left panel (*p* < 0.0001) followed by Dunn's multiple comparisons test (Control#1 vs ASD#4, **p* < 0.05, Control#2 vs ASD#4, *****p* < 0.0001, Control#3 vs ASD#4, *****p* < 0.0001, Control#3 vs ASD#5, **p* < 0.05); Mann-Whitney test, ***p* < 0.01]. (d) A significant increase in the number of action potentials (AP) was observed in ASD iN cells [Control: n = 64, ASD: n = 135 (Control#1: n= 45, Control#2: n= 30, Control#3: n= 19, ASD#3: n= 62, ASD#4: n= 49, ASD#5: n= 24); two-way repeated-measures ANOVA, interaction, F (6, 1722) = 1.995, *p* = 0.0633; Bonferroni post hoc test, 50mV, **p* = 0.0270, 60mV, ****p* = 0.0005]. (e) AP threshold was comparable between control and ASD iN cells [Control: n = 85, ASD: n = 98 (Control#1: n= 46, Control#2: n= 22, Control#3: n= 17, ASD#3: n= 35, ASD#4: n= 35, ASD#5: n= 28), one-way ANOVA for left panel (interaction, *p* < 0.0001, F_(5, 177)_ = 7.400) followed by Bonferroni post hoc test (Control#1 vs ASD#5, **p* < 0.05, Control#3 vs ASD#3, *****p* < 0.0001, Control#3 vs ASD#4, ***p* < 0.01); unpaired *t*-test, *p* = 0.8458]. (f) ASD iN cells showed an increase in AP amplitude [Control: n = 84, ASD: n = 97 (Control#1: n= 45, Control#2: n= 22, Control#3: n= 17, ASD#3: n= 35, ASD#4: n= 35, ASD#5: n= 27), Kruskal-Wallis test for left panel (*p* < 0.0001) followed by Dunn's multiple comparisons test (Control#2 vs ASD#4, ***p* < 0.01, Control#2 vs ASD#5, **p* < 0.05, Control#3 vs ASD#4, ****p* < 0.001, Control#3 vs ASD#5, ***p* < 0.01); unpaired *t*-test, *p* = 0.0036). (g) Significant decrease in AP half-width was detected in the ASD iN cells [Control: n = 74, ASD: n = 96 (Control#1: n= 44, Control#2: n= 19, Control#3: n= 11, ASD#3: n= 34, ASD#4: n= 35, ASD#5: n= 27), Kruskal-Wallis test for left panel (*p* < 0.001) followed by Dunn's multiple comparisons test (Control#3 vs ASD#3, **p* < 0.05, Control#3 vs ASD#4, ****p* < 0.001, Control#3 vs ASD#5, ***p* < 0.01); Mann-Whitney test, ***p* < 0.01). (h) No changes were observed in frequency of mEPSC [Control: n = 58, ASD: n = 85 (Control#1: n= 35, Control#2: n= 12, Control#3: n= 11, ASD#3: n= 49, ASD#4: n= 18, ASD#5: n= 18), Kruskal-Wallis test for left panel (*p* < 0.05) followed by Dunn's multiple comparisons test; Mann-Whitney test, *p* = 0.0600]. (i) Increased mEPSC amplitude was detected in ASD iN cells [Control: n = 58, ASD: n = 85 (Control#1: n= 35, Control#2: n = 12, Control#3: n= 11, ASD#3: n= 49, ASD#4: n= 18, ASD#5: n= 18), Kruskal-Wallis test for left panel (*p* < 0.05) followed by Dunn's multiple comparisons test (Control#2 vs ASD#3, ***p* < 0.01); Mann-Whitney test, **p* < 0.05].

**
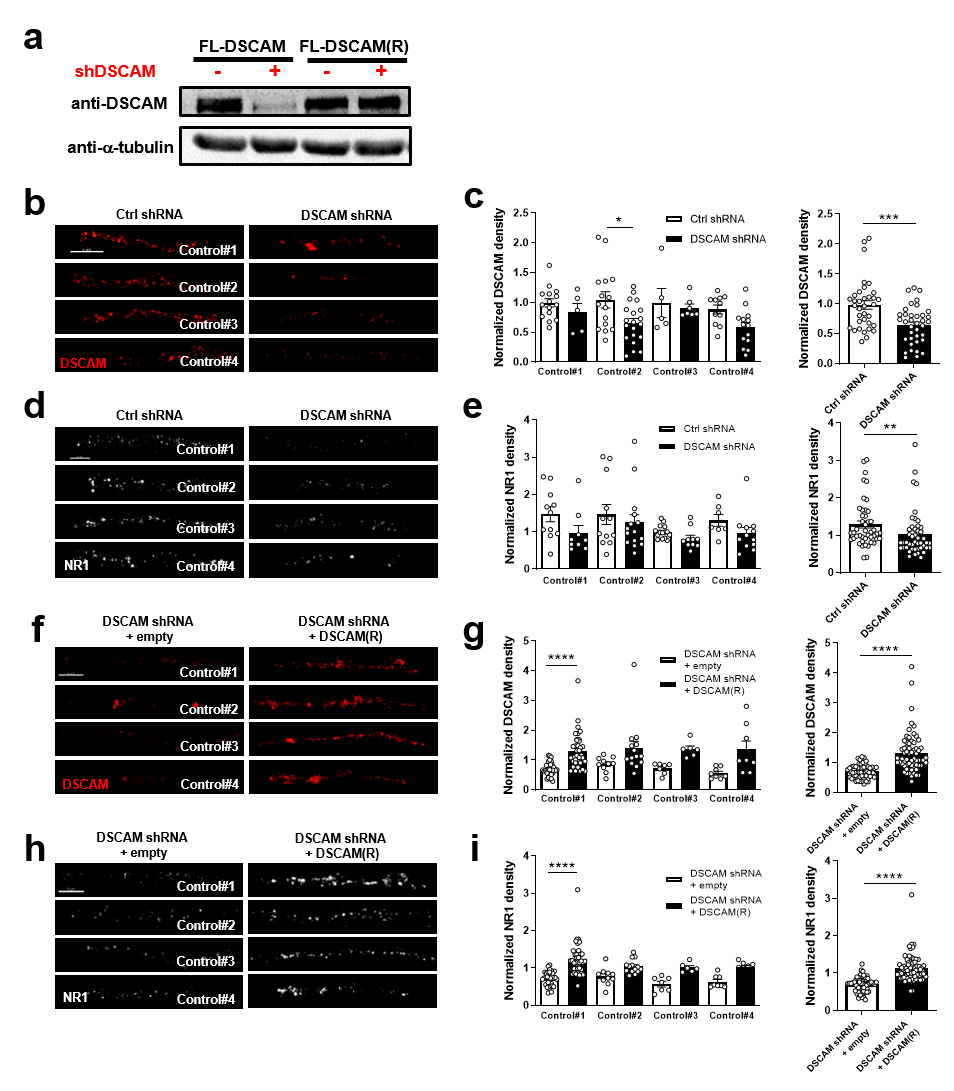
**

**Supplementary Figure 5. DSCAM density and NR1 density in iN cells.**

(a) Western blot analysis of the knock-down efficiency of a DSCAM shRNA. Expression of full-length DSCAM (but not of shRNA-resistant DSCAM [DSCAM(R)]) was clearly reduced using a DSCAM-specific shRNA in LX293T cells. (b-e) Knock-down of endogenous DSCAM expression using a specific DSCAM shRNA led to significantly decreased DSCAM and NR1 density in control iN cells. Representative immunocytochemical images of DSCAM (b) and NR1 expression (d) and quantitative analysis of normalized DSCAM (c) and NR1 density (e) in control iN cells after control (Ctrl) or DSCAM shRNA expression. (DSCAM density-Ctrl shRNA: n = 46; Control#1: n = 15, Control#2: n = 15, Control#3: n = 5, Control#4: n = 11, DSCAM shRNA: n = 47; Control#1: n = 5, Control#2: n = 20, Control#3: n = 7, Control#4: n = 15, one-way ANOVA for left panel, *p* < 0.01, F_(7, 84)_ = 3.1, Tukey’s multiple comparisons test (Control#2 + control shRNA vs Control#2 + DSCAM shRNA, **p* < 0.05), unpaired *t*-test for right panel, ****p* < 0.001) (NR1 density-Control shRNA: n = 46; Control#1: n = 15, Control#2: n = 15, Control#3: n = 5, Control#4: n = 11, DSCAM shRNA: n = 46; Control#1: n = 5, Control#2: n = 20, Control#3: n = 7, Control#4: n = 14, Kruskal-Wallis test for left panel, *p* <0.05, Dunn’s multiple comparisons test, ns, not significant, Mann-Whitney test for right panel, ***p* < 0.01). Scale bar: 5 μm. (f-g) Exogenous expression of shRNA-resistant full-length DSCAM [DSCAM(R)] combined with a shRNA against endogenous DSCAM in control iN cells rescued DSCAM density. (f) Representative immunocytochemical images. Scale bar: 5 μm. (g) Quantitative analysis of DSCAM expression in control iN cells after expressing DSCAM shRNA only or DSCAM shRNA combined with full-length DSCAM(R). (DSCAM shRNA + empty: n = 60; Control#1: n = 33, Control#2: n = 10, Control#3: n = 7, Control#4: n = 10, DSCAM shRNA + DSCAM(R): n =70; Control#1: n = 38, Control#2: n = 14, Control#3: n = 6, Control#4: n = 12). Kruskal-Wallis test for left panel, *p* <0.0001, Dunn’s multiple comparisons test, Control#1 + DSCAM shRNA + empty vs Control#1 + DSCAM shRNA + DSCAM(R), *****p* < 0.0001, Mann-Whitney test for right panel, *****p* < 0.0001. (h-i) Exogenous expression of shRNA-resistant full-length DSCAM [DSCAM(R)] combined with a shRNA against endogenous DSCAM in control iN cells rescued NR1 density. (h) Representative immunocytochemical images. Scale bar: 5 μm. (i) Quantitative analysis of NR1 expression in control iN cells after expressing *DSCAM* shRNA only or *DSCAM* shRNA combined with full-length DSCAM(R) expression. (DSCAM shRNA + empty: n = 60; Control#1: n = 33, Control#2: n = 10, Control#3: n = 7, Control#4: n = 10, DSCAM shRNA + DSCAM(R): n = 70; Control#1: n = 38, Control#2: n = 14, Control#3: n = 6, Control#4: n = 12). Kruskal-Wallis test for left panel, *p* <0.0001, Dunn’s multiple comparisons test, Control#1 + DSCAM shRNA + empty vs Control#1 + DSCAM shRNA + DSCAM(R), *****p* < 0.0001, Mann-Whitney test for right panel, *****p* < 0.0001.

**
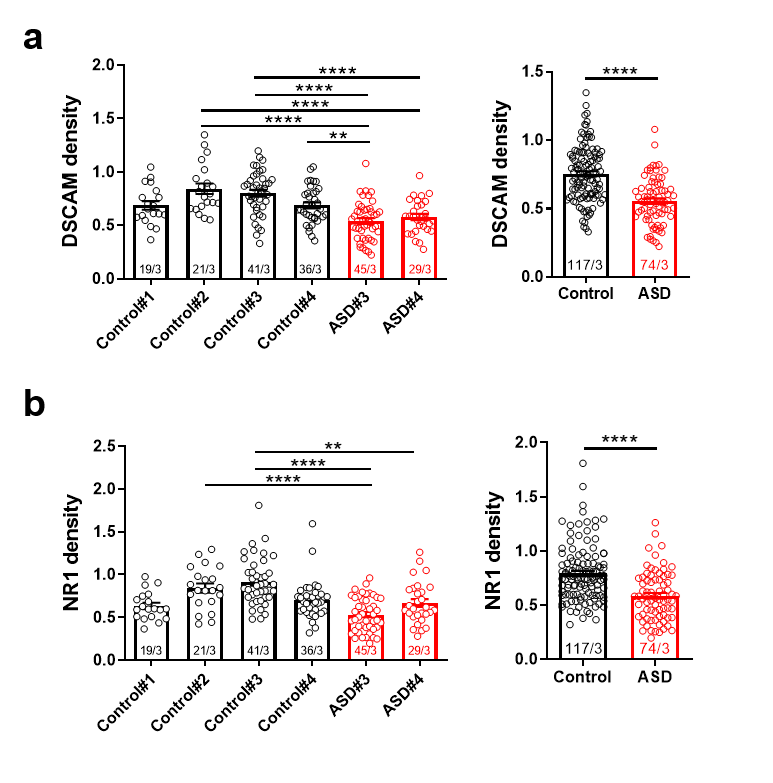
**

**Supplementary Figure 6. DSCAM density and NR1 density in iN cells.**

(a) ASD iN cells had significantly lower DSCAM density as compared to control iN cells [Control: n = 117, ASD: n = 74 (Control#1: n= 19, Control#2: n= 21, Control#3: n= 41, Control#4: n= 36, ASD#3: n= 45, ASD#4: n= 29), one-way ANOVA for left panel (interaction, *p* < 0.0001, F_(5, 185)_ = 13.36) followed by Bonferroni post hoc test (Control#2 vs ASD#3, *****p* < 0.0001, Control#2 vs ASD#4, *****p* < 0.0001, Control#3 vs ASD#3, *****p* < 0.0001, Control#3 vs ASD#4, *****p* < 0.0001, Control#4 vs ASD#3, ***p* < 0.01); unpaired *t*-test for right panel, *****p* < 0.0001]. (b) Lower NR1 density was detected in ASD iN cells [Control: n = 117, ASD: n = 74 (Control#1: n= 19, Control#2: n= 21, Control#3: n= 41, Control#4: n= 36, ASD#3: n= 45, ASD#4: n= 29), Kruskal-Wallis test for left panel (*p* < 0.0001) followed by Dunn's multiple comparisons test (Control#2 vs ASD#3, *****p* < 0.0001, Control#3 vs ASD#3, *****p* < 0.0001, Control#3 vs ASD#4, ***p* < 0.01); Mann-Whitney test for right panel, *****p* < 0.0001].

**
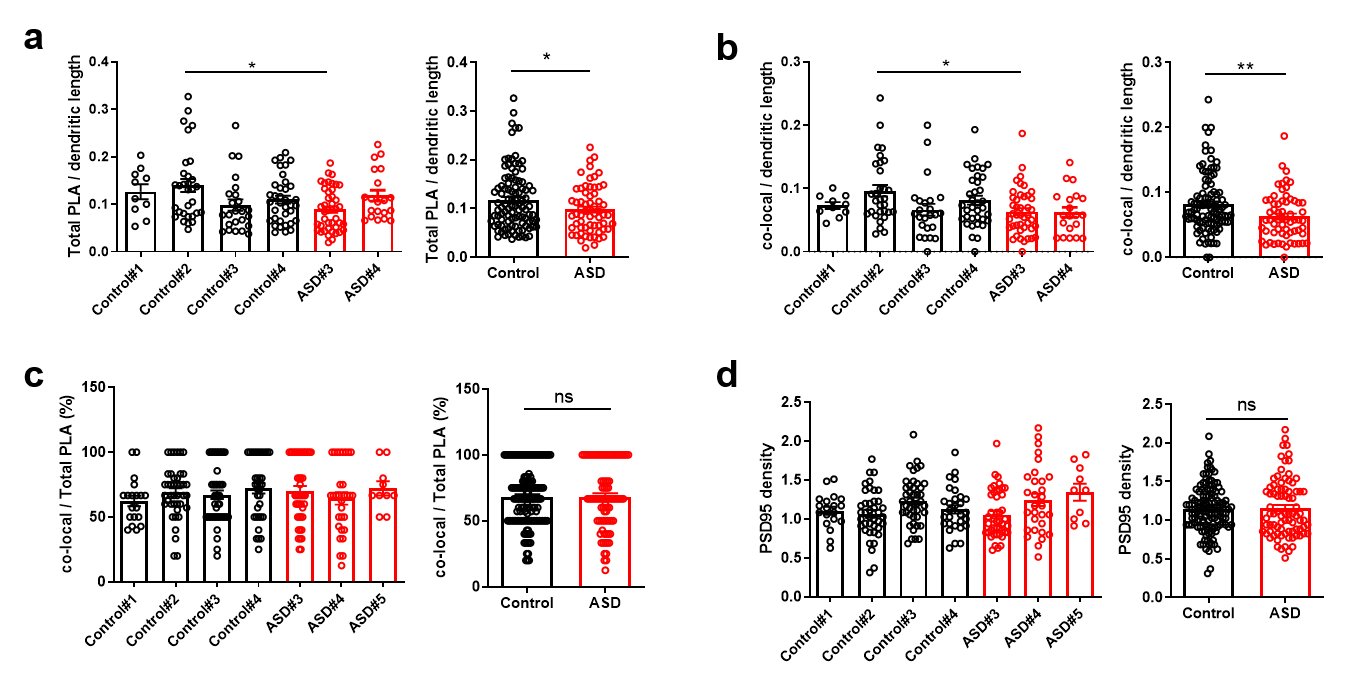
**

**Supplementary Figure 7. Quantitative analysis of PLA signals.**

(a) Quantitative analysis of normalized total PLA signals resulted in a significant decrease in ASD iN cells [Control: n = 101, ASD: n = 63 (Control#1: n = 10, Control#2: n = 31, Control#3: n = 24, Control#4: n = 36, ASD#3: n = 43, ASD#4: n = 20), Kruskal-Wallis test for left panel (*p* < 0.05) followed by Dunn's multiple comparisons test (Control#2 vs ASD#3, **p* < 0.05), One-tailed Mann-Whitney test for right panel (**p* = 0.0383)]. Quantification of total PLA was calculated as the number of total PLA divided by the length of neurite (µm). (b) Co‑localization between PLA signals and PSD-95 puncta also decreased in ASD iN cells. [Control: n = 101, ASD: n = 63 (Control#1: n = 10, Control#2: n = 31, Control#3: n = 24, Control#4: n = 36, ASD#3: n = 43, ASD#4: n = 20), Kruskal-Wallis test for left panel (*p* < 0.05) followed by Dunn's multiple comparisons test (Control#2 vs ASD#3, **p* < 0.05), Mann-Whitney test for right panel (***p* < 0.01)]. Quantification of co-localization of PLA signals and PSD-95 puncta was evaluated as the number of co-localized puncta divided by the length of neurite (µm). (c) The percent of the co-localization between PLA signals and PSD-95 puncta per the number of the total PLA was not changed between control and ASD iN cells [Control: n = 142, ASD: n = 85 (Control#1: n = 19, Control#2: n = 41, Control#3: n = 47, Control#4: n = 35, ASD#3: n = 44, ASD#4: n = 31, ASD#5: n= 10), Kruskal-Wallis test for left panel, ns, not significant, Mann-Whitney test for right panel, ns, not significant]. (d) Quantitative analysis of normalized PSD95 showed similar density in both control and ASD iN cells. [Control: n = 132, ASD: n = 84 (Control#1: n = 19, Control#2: n = 40, Control#3: n = 44, Control#4: n = 29, ASD#3: n = 44, ASD#4: n = 30, ASD#5: n = 10), one-way ANOVA for left panel, *p* < 0.05, F_(6, 209)_ = 2.726, Mann-Whitney test for right panel ns, not significant].

**
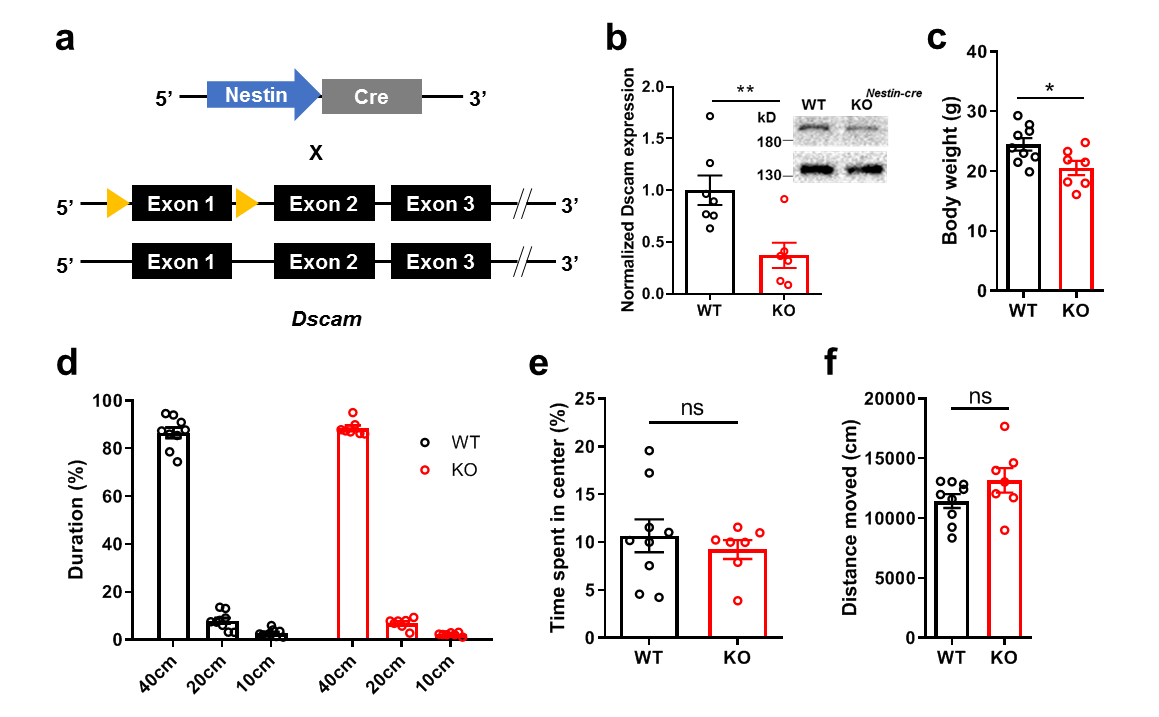
Supplementary Figure 8. Generation and characterization of neural stem cell-specific *Dscam* KO mice**

(a) Schematic view of the generation of heterozygous Nestin-*Dscam* knockout (KO) mice. Heterozygous *Dscam* floxed exon 1 mice were crossed with Nestin-Cre mice. (b) Normalized Dscam expression was reduced in the anterior cingulate cortex (ACC) of Nestin-*Dscam* KO mice (wild-type [WT]: n = 7, KO: n = 6, unpaired *t*-test, ***p* < 0.01). (inset) Representative Western blot images. (c) Body weight was significantly reduced in Nestin-*Dscam* KO mice as compared to WT littermates (WT: n = 9, KO: n = 7, unpaired *t*-test, **p* < 0.05). (d-f) The open-field test was conducted to examine the locomotive activity and anxiety level of Nestin-*Dscam* KO mice. (d) Percent of time spent in the 40 cm, 20 cm, or 10 cm zones of the open field (WT: n = 9, KO: n = 7). (e) Percent of time spent in the center zone (Mann-Whitney test, ns, not significant). (f) The distance that Nestin-*Dscam* KO mice moved in the open field was similar to WT littermates (unpaired *t*-test, ns, not significant).

**
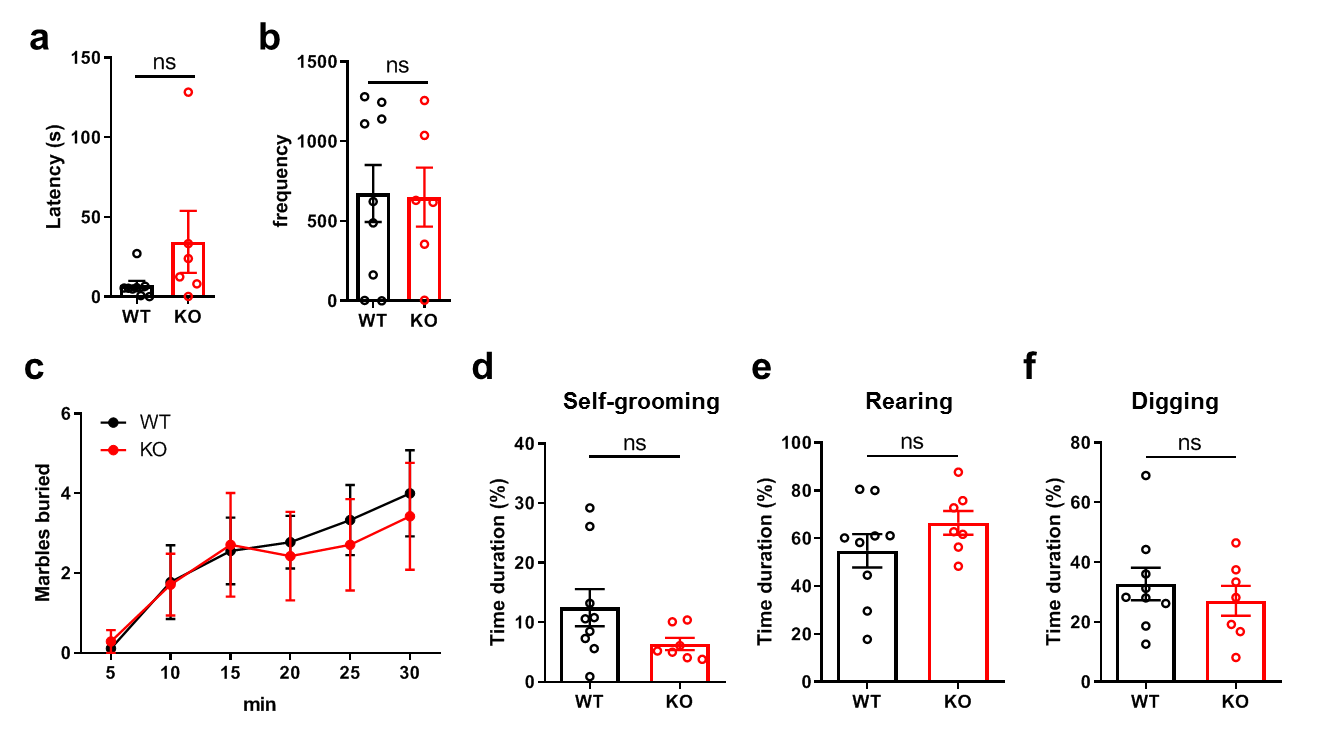
 Supplementary Figure 9. No changes in social communication and repetitive behaviors in Nestin-*Dscam* KO mice.**

(a-b) An ultrasonic vocalization test was conducted to examine the social communication rate. (a) The latency to first call of Nestin-*Dscam* knockout (KO) mice did not differ from wild-type (WT) littermates (WT: n = 8, KO: n = 6, Mann-Whitney test, ns, not significant). (b) The frequency of calls in Nestin-*Dscam* KO mice was comparable to WT littermates (unpaired *t*-test, ns, not significant). (c) The marble burying test was performed to evaluate repetitive behaviors of Nestin-*Dscam* KO mice. The number of marbles buried by Nestin-*Dscam* KO mice was comparable to that of WT littermates (WT: n = 9, KO: n = 7). (d-f) Duration of repetitive behaviors, such as self-grooming (unpaired *t*-test, ns, not significant), rearing (unpaired *t*-test, ns, not significant), and digging behaviors (Mann-Whitney test, ns, not significant), did not differ between Nestin-*Dscam* KO mice and WT littermates (WT: n = 9, KO: n = 7).

**Supplementary Table 1. List of iPSC lines used**

| **Information** | **Gender** | **Age** | **Origin** | **Disease** | **Genera-tion** | **Reprogramming  methodology** |  | **Ethnicity** |
| --- | --- | --- | --- | --- | --- | --- | --- | --- |
|  |  |  |  |  |  |  |  |  |
| Control#1 | Female | 37 | fibroblast | Normal | Yes | Episomal iPSC reprogramming plasmids/Electroporation |  | Asian |
| Control#2 | Male | 5 | foreskin | Normal | Yes | Episomal iPSC reprogramming plasmids/Electroporation |  | Asian |
| Control#3 | Male | 8 | fibroblast | Normal | Yes | Episomal iPSC reprogramming plasmids/Electroporation |  | Asian |
| Control#4  [CRL-2097™]^1^ | Male | newborn | fibroblast | Normal | Yes^1^ | Episomal iPSC reprogramming plasmids/Electroporation |  | - |
|  |  |  |  |  |  |  |  |  |
| ASD# (3,4,5) | Male | 12 | fibroblast | ASD | Yes | Episomal iPSC reprogramming plasmids/Electroporation |  | Asian |

1. [Jung KB](https://www.ncbi.nlm.nih.gov/pubmed/?term=Jung%20KB%5BAuthor%5D&cauthor=true&cauthor_uid=30072687), [Lee H](https://www.ncbi.nlm.nih.gov/pubmed/?term=Lee%20H%5BAuthor%5D&cauthor=true&cauthor_uid=30072687), [Son YS](https://www.ncbi.nlm.nih.gov/pubmed/?term=Son%20YS%5BAuthor%5D&cauthor=true&cauthor_uid=30072687), [Lee MO](https://www.ncbi.nlm.nih.gov/pubmed/?term=Lee%20MO%5BAuthor%5D&cauthor=true&cauthor_uid=30072687), [Kim YD](https://www.ncbi.nlm.nih.gov/pubmed/?term=Kim%20YD%5BAuthor%5D&cauthor=true&cauthor_uid=30072687), [Oh SJ](https://www.ncbi.nlm.nih.gov/pubmed/?term=Oh%20SJ%5BAuthor%5D&cauthor=true&cauthor_uid=30072687), [Kwon O](https://www.ncbi.nlm.nih.gov/pubmed/?term=Kwon%20O%5BAuthor%5D&cauthor=true&cauthor_uid=30072687), [Cho S](https://www.ncbi.nlm.nih.gov/pubmed/?term=Cho%20S%5BAuthor%5D&cauthor=true&cauthor_uid=30072687), [Cho HS](https://www.ncbi.nlm.nih.gov/pubmed/?term=Cho%20HS%5BAuthor%5D&cauthor=true&cauthor_uid=30072687), [Kim DS](https://www.ncbi.nlm.nih.gov/pubmed/?term=Kim%20DS%5BAuthor%5D&cauthor=true&cauthor_uid=30072687), [Oh JH](https://www.ncbi.nlm.nih.gov/pubmed/?term=Oh%20JH%5BAuthor%5D&cauthor=true&cauthor_uid=30072687), [Zilbauer M](https://www.ncbi.nlm.nih.gov/pubmed/?term=Zilbauer%20M%5BAuthor%5D&cauthor=true&cauthor_uid=30072687), [Min JK](https://www.ncbi.nlm.nih.gov/pubmed/?term=Min%20JK%5BAuthor%5D&cauthor=true&cauthor_uid=30072687), [Jung CR](https://www.ncbi.nlm.nih.gov/pubmed/?term=Jung%20CR%5BAuthor%5D&cauthor=true&cauthor_uid=30072687), [Kim J](https://www.ncbi.nlm.nih.gov/pubmed/?term=Kim%20J%5BAuthor%5D&cauthor=true&cauthor_uid=30072687), [Son MY](https://www.ncbi.nlm.nih.gov/pubmed/?term=Son%20MY%5BAuthor%5D&cauthor=true&cauthor_uid=30072687). Interleukin-2 induces the in vitro maturation of human pluripotent stem cell-derived intestinal organoids [Nat Commun.](https://www.ncbi.nlm.nih.gov/pubmed/?term=Jung%2C+Kwang+Bo%2C+et+al.+%22Interleukin-2+induces+the+in+vitro+maturation+of+human+pluripotent+stem+cell-derived+intestinal+organoids) 2018 Aug 2;9(1):3039. doi: 10.1038/s41467-018-05450-8.

**Supplementary Table 2. Characterization of iPSC lines generated**

| **Characterization** | **AP staining** | **Immuno- cytochemistry** | **Real-time  qPCR** | **Mutation  check** |
| --- | --- | --- | --- | --- |
|  |  |  |  |  |
| Control#1 | Yes | Yes | Yes | Yes |
| Control#2 | Yes | Yes | Yes | Yes |
| Control#3 | Yes | Yes | Yes | Yes |
| Control#4  [CRL-2097™]^1^ | - | - | - | Yes |
|  |  |  |  |  |
| ASD#3 | Yes | Yes | Yes | Yes |
| ASD#4 | Yes | Yes | Yes | Yes |
| ASD#5 | Yes | Yes | Yes | Yes |

1. [Jung KB](https://www.ncbi.nlm.nih.gov/pubmed/?term=Jung%20KB%5BAuthor%5D&cauthor=true&cauthor_uid=30072687), [Lee H](https://www.ncbi.nlm.nih.gov/pubmed/?term=Lee%20H%5BAuthor%5D&cauthor=true&cauthor_uid=30072687), [Son YS](https://www.ncbi.nlm.nih.gov/pubmed/?term=Son%20YS%5BAuthor%5D&cauthor=true&cauthor_uid=30072687), [Lee MO](https://www.ncbi.nlm.nih.gov/pubmed/?term=Lee%20MO%5BAuthor%5D&cauthor=true&cauthor_uid=30072687), [Kim YD](https://www.ncbi.nlm.nih.gov/pubmed/?term=Kim%20YD%5BAuthor%5D&cauthor=true&cauthor_uid=30072687), [Oh SJ](https://www.ncbi.nlm.nih.gov/pubmed/?term=Oh%20SJ%5BAuthor%5D&cauthor=true&cauthor_uid=30072687), [Kwon O](https://www.ncbi.nlm.nih.gov/pubmed/?term=Kwon%20O%5BAuthor%5D&cauthor=true&cauthor_uid=30072687), [Cho S](https://www.ncbi.nlm.nih.gov/pubmed/?term=Cho%20S%5BAuthor%5D&cauthor=true&cauthor_uid=30072687), [Cho HS](https://www.ncbi.nlm.nih.gov/pubmed/?term=Cho%20HS%5BAuthor%5D&cauthor=true&cauthor_uid=30072687), [Kim DS](https://www.ncbi.nlm.nih.gov/pubmed/?term=Kim%20DS%5BAuthor%5D&cauthor=true&cauthor_uid=30072687), [Oh JH](https://www.ncbi.nlm.nih.gov/pubmed/?term=Oh%20JH%5BAuthor%5D&cauthor=true&cauthor_uid=30072687), [Zilbauer M](https://www.ncbi.nlm.nih.gov/pubmed/?term=Zilbauer%20M%5BAuthor%5D&cauthor=true&cauthor_uid=30072687), [Min JK](https://www.ncbi.nlm.nih.gov/pubmed/?term=Min%20JK%5BAuthor%5D&cauthor=true&cauthor_uid=30072687), [Jung CR](https://www.ncbi.nlm.nih.gov/pubmed/?term=Jung%20CR%5BAuthor%5D&cauthor=true&cauthor_uid=30072687), [Kim J](https://www.ncbi.nlm.nih.gov/pubmed/?term=Kim%20J%5BAuthor%5D&cauthor=true&cauthor_uid=30072687), [Son MY](https://www.ncbi.nlm.nih.gov/pubmed/?term=Son%20MY%5BAuthor%5D&cauthor=true&cauthor_uid=30072687). Interleukin-2 induces the in vitro maturation of human pluripotent stem cell-derived intestinal organoids [Nat Commun.](https://www.ncbi.nlm.nih.gov/pubmed/?term=Jung%2C+Kwang+Bo%2C+et+al.+%22Interleukin-2+induces+the+in+vitro+maturation+of+human+pluripotent+stem+cell-derived+intestinal+organoids) 2018 Aug 2;9(1):3039. doi: 10.1038/s41467-018-05450-8.

**Supplementary Table 3.** **Primer sequences for qRT-PCR**

| **Gene name** | **Forward (5’🡪3’)** | **Reverse (5’🡪3’)** |
| --- | --- | --- |
| hDscam | TTGCGGTCTTCAAGTGCATTA | TGCAGCGGTAGTTATACAATCCA |
| GRIN1 | ACCCCAAGATCGTCAACATTG | GGCTAACTAGGATGGCGTAGA |
| GRIN2A | TGGCCTCACCGGGTATGATT | CAATGCCGTCCCTCACTCTC |
| GRIN2B | GTCCCTGGACGATGGAGATTC | CAGTCAGCCCTACTGAGTTGG |
| LRRTM1 | TCGGGCAACGAGATCGAGTA | GCTTGTCAGGGACTTCCAAGA |
| SYT6 | CTCCCGCCATGACATGATTG | TCCCAAGTCCACGCTTTCAC |
| Sox2 | GGGAAATGGGAGGGGTGCAAAAGAGG | TTGCGTGAGTGTGGATGGGATTGGTG |
| Nanog | AAGACAAGGTCCCGGTCAAG | CAGGCATCCCTGGTGGTAG |
| Rex1 | AAGGCAAGTCAAGCCAAGACC | TTCCAAAGAACATTCAAGGGAGC |
| Oct3/4 | CCCCAGGGCCCCATTTTGGTACC | ACCTCAGTTTGAATGCATGGGAGAGC |
